# Polyphenol‐Mediated Engineering of Lipid Nanoparticles With Crystalline Mesophases

**DOI:** 10.1002/adma.202505830

**Published:** 2025-09-15

**Authors:** Shiyao Li, Patrick Charchar, Haitao Yu, Zhixing Lin, Hamish G. Brown, Wanjun Xu, Chan‐Jin Kim, Irene Yarovsky, Yi Ju, Frank Caruso

**Affiliations:** ^1^ Department of Chemical Engineering The University of Melbourne Parkville Victoria 3010 Australia; ^2^ School of Science RMIT University Melbourne Victoria 3000 Australia; ^3^ Olivia Newton‐John Cancer Research Institute and School of Cancer Medicine La Trobe University Heidelberg Victoria 3084 Australia; ^4^ School of Engineering RMIT University Melbourne Victoria 3000 Australia; ^5^ Ian Holmes Imaging Centre Bio21 Molecular Science and Biotechnology Institute University of Melbourne Parkville Victoria 3052 Australia

**Keywords:** drug delivery, nanostructures, nonlamellar mesophases, polyphenols, self‐assembly

## Abstract

Lipids self‐assemble into lipid nanoparticles (LNPs) with different crystalline mesophases, including lamellar and nonlamellar (e.g., cubic and hexagonal) mesophases. Although various additives can modulate lipid curvature, the formation of nonlamellar crystalline mesophases in LNPs typically depends on specific phase‐forming lipids, which limits the overall design space and structural versatility for cargo loading. Herein, a new class of nonlamellar LNPs is engineered through the one‐step assembly of two components—a polyphenol and a lipid—where the lipid mesophases are modulated by the polyphenols in a concentration‐dependent manner. The polyphenol‐based LNPs exhibit ordered Im3m micellar cubic or hexagonal nanostructures and can load metal ions, small‐molecule drugs, polypeptides, proteins, polysaccharides, and mRNA. The present study establishes an approach to generate nonlamellar LNPs with ordered nanostructures and functional cargos for diverse applications.

## Graphical abstract

A polyphenol‐mediated strategy enables the one‐step formation of lipid nanoparticles with nonlamellar mesophases, including micellar cubic and hexagonal structures. These highly ordered nanostructures accommodate a wide range of cargos—from metal ions to mRNA—and offer a modular and versatile platform for constructing functional lipid‐based delivery systems with tunable internal order and potential for diverse applications.

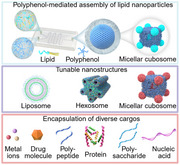

## 1 Introduction

Lipids are essential biomolecules that not only form the structural foundation of cellular membranes,^[^ ^1^ ^]^ but also regulate a wide range of biological processes, including intracellular signalling,^[^ ^2^ ^]^ energy storage,^[^ ^3^ ^]^ and molecular recognition.^[^ ^4^ ^]^ Lipids possess an intrinsic ability to self‐assemble into a range of liquid crystalline mesophases—including lamellar,^[^ ^5^ ^]^ and curvature‐dependent Type I (normal) and Type II (inverse) micellar, micellar cubic, hexagonal, and bicontinuous cubic phases^[^ ^6^, ^7^, ^8^ ^]^—depending on their molecular geometry. This structural diversity is largely governed by the critical packing parameter (CPP), which relates molecular shape to interfacial curvature.^[^ ^9^, ^10^, ^11^, ^12^ ^]^ Those lipid assemblies in different mesophases have been shown to mediate molecular transport and biochemical reactions in biological systems,^[^ ^13^, ^14^ ^]^ and have inspired the development of lipid‐based nanocarriers, e.g., liposomes, as well as Type I and Type II cubosomes (including micellar and bicontinuous) and hexosomes.^[^ ^11^ ^]^

Lipid‐based nanocarriers have been widely explored for drug delivery applications, exemplified by Doxil^[^ ^15^ ^]^ (the first liposomal formulation of doxorubicin (DOX) approved by the US Food and Drug Administration), as well as Onpattro^[^ ^16^ ^]^ and mRNA COVID‐19 vaccines (BioNTech/Pfizer's BNT162b2 and Moderna's mRNA‐1273),^[^ ^17^ ^]^ which incorporate ionizable lipids into the traditional liposomal formulation to facilitate RNA encapsulation. Compared with liposomes, nonlamellar liquid crystalline lipid nanoparticles (LC‐LNPs) (e.g., cubosomes and hexosomes) exhibit higher surface areas for the encapsulation of hydrophilic or hydrophobic molecules due to their internally curved and ordered nanostructures.^[^ ^11^, ^18^, ^19^, ^20^ ^]^

However, the current engineering strategies of LC‐LNPs primarily rely on the self‐guided phase behaviors of limited types of lipids that intrinsically adopt nonlamellar mesophases, e.g., monoolein (MO) and phytantriol (PHY)),^[^ ^18^, ^21^ ^]^ and synthetic amphiphiles which require precise molecular design.^[^ ^22^ ^]^ Furthermore, the stability and structural integrity of the resulting nonlamellar mesophases are influenced by multiple factors, e.g., temperature,^[^ ^23^ ^]^ storage time,^[^ ^11^ ^]^ and the presence of guest molecules,^[^ ^10^ ^]^ restricting their scalability and broader applications.

In this study, we establish a one‐step assembly strategy for engineering stable LC‐LNPs by tuning the lipid mesophase through interactions with polyphenols, e.g., tannic acid (TA), which enable the formation of liquid crystalline structures not accessible by the lipids alone. Experimental results and all‐atom molecular dynamics (MD) simulations reveal that increasing the concentration of TA affords the formation of *Im3m* micellar cubic mesophase in the LNPs. The liquid crystalline nanostructure, formed through hydrophobic interactions of lipid tails and interactions between lipid head groups and polyphenols, remains stable under storage for over a year. A range of polyphenol‐based LC‐LNPs (PLC‐LNPs) with tunable sizes (90–420 nm), lattice parameters (106–151Å), and different nanostructures (multilamellar and hexagonal) are engineered by using different assembly components. Furthermore, the formation of the cubic nanostructure is not influenced by the incorporation of guest molecules, i.e., metal ions, small‐molecule drugs, polypeptides, proteins, polysaccharides, and mRNA (molecular weights range from 200 Da to 300 kDa), making them promising for potential application in diverse fields.

## 2 Results and Discussion

### 2.1 Polyphenol‐Mediated Assembly of LC‐LNPs

**Figure 1:**
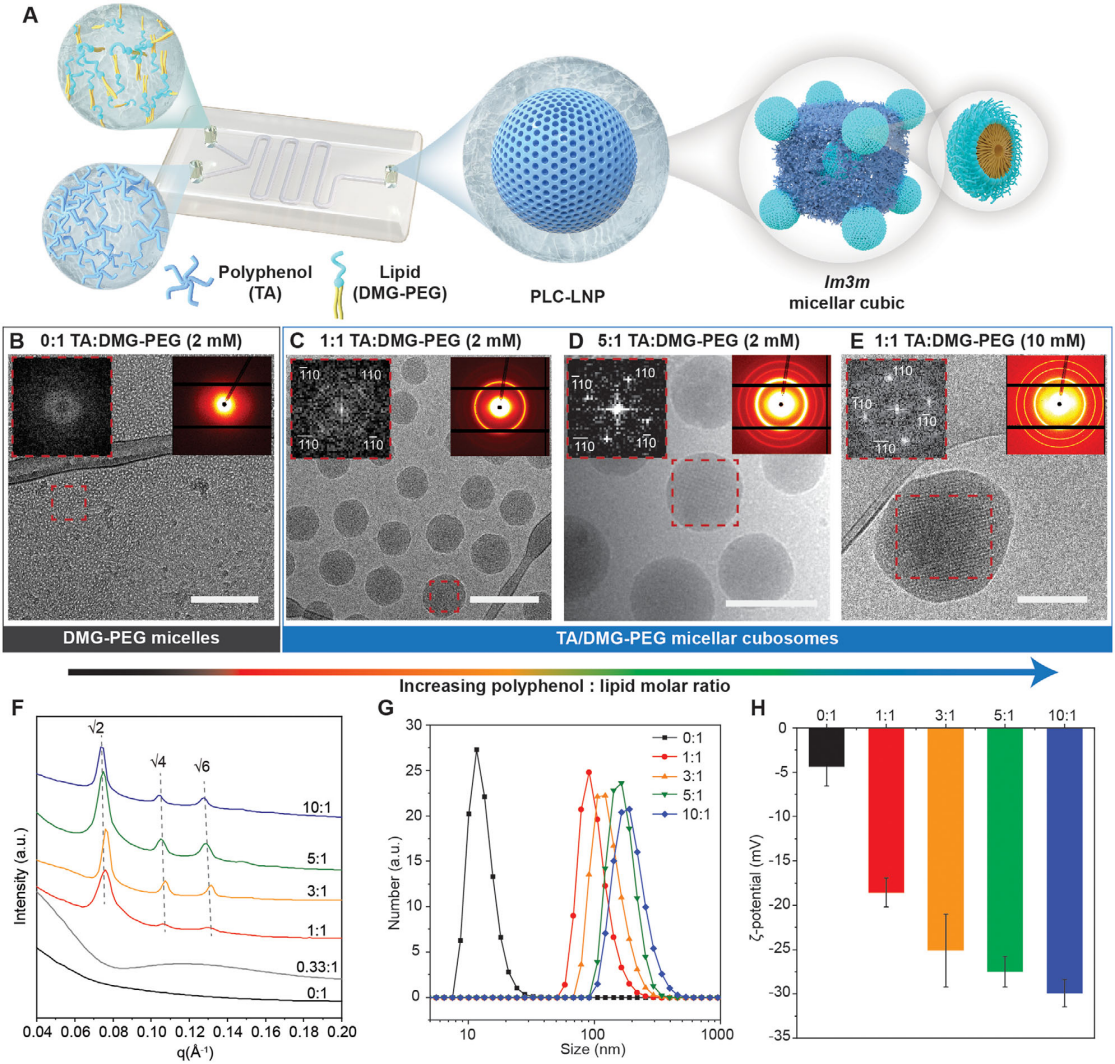
Self‐assembly of TA/DMG‐PEG PLC‐LNPs. A) Schematic of the preparation of TA/DMG‐PEG micellar cubosomes using a NanoAssemblr microfluidic device. B–E) Cryo‐transmission electron microscopy images of DMG‐PEG NPs (B) and TA/DMG‐PEG micellar cubosomes assembled using different concentrations of TA and DMG‐PEG (formulations are listed in Table S1, Supporting Information) C–E). Scale bars are 200 nm. Insets are fast Fourier transform images with assigned Miller indices for the section marked with a red frame (left) and 2D SAXS patterns of the samples (right). F–H) 1D SAXS patterns (F), size distribution (G), and ζ‐potential (H) of DMG‐PEG (0:1) and TA/DMG‐PEG NPs assembled using increasing polyphenol (TA)‐to‐lipid (DMG‐PEG) molar ratios from 0:1 to 10:1. The concentration of DMG‐PEG used for LNP preparation was 2 mm. In (H), data are shown as mean ± standard deviation (SD) (*n* = 3 independent replicates).

Mixing polyphenols (TA dissolved in aqueous solution) and lipids (1,2‐dimyristoyl‐*rac*‐glycero‐3‐methoxypolyethylene glycol‐2000 (DMG‐PEG) dissolved in ethanol) using a microfluidic device (Ignite NanoAssemblr) resulted in the self‐assembly of LC‐LNPs with *Im3m* micellar cubic mesophase (**Figure** **1**A). The mesophase of the LNPs was dependent on the molar ratio of TA and DMG‐PEG. In the absence of TA, DMG‐PEG lipid (2 mm) self‐assembled into Type I micelles without ordered nanostructures (Figure 1B; Table S1, Supporting Information), which is consistent with previous studies that PEG‐lipids self‐assemble into micelles due to their high positive curvature.^[^ ^24^, ^25^, ^26^ ^]^ When the TA‐to‐DMG‐PEG molar ratio was 0.33:1, the broad peak in the small‐angle X‐ray scattering (SAXS) pattern of the NPs indicated an emerging disordered micellar cubic phase (Figure 1F), which could be attributed to TA binding with DMG‐PEG micelles and reduction of its positive lipid interfacial curvature. Monodisperse *Im3m* micellar cubosomes (referred to as cubosomes hereafter) with relative positions in the spacing ratios of √2:√4:√6 in the SAXS pattern formed when the TA‐to‐DMG‐PEG molar ratio was equal to or higher than 1:1 (Figure 1C,D,F; Figure S1 and Table S2, Supporting Information). Mesophase transition in the TA/DMG‐PEG NPs as a function of polyphenol‐to‐lipid ratio followed a similar trend when a higher concentration of DMG‐PEG (10 mm) was used for NP preparation (Figure 1E; Figure S2, Supporting Information). The size of the TA/DMG‐PEG cubosomes, which was dependent on the concentrations of the polyphenols and lipids, could be tuned from 90 to 260 nm (Figure 1C–E,G; Table S1, Supporting Information). Increasing the TA‐to‐DMG‐PEG molar ratio from 0:1 to 10:1 resulted in decreasing ζ‐potentials of NPs from −4 to −30 mV, suggesting the presence of TA on the cubosome surface (Figure 1H; Table S1, Supporting Information). From the ^1^H nuclear magnetic resonance (^1^H NMR) spectra of TA/DMG‐PEG cubosomes, increasing the TA‐to‐DMG‐PEG molar ratio also led to a higher proportion of TA in the cubosomes (Figure S3, Supporting Information). TA/DMG‐PEG cubosomes disassembled in Tween 20 but remained stable in NaCl and urea solution (Figures S4 and S5, Supporting Information), suggesting that the dominant interactions involved in the NP assembly are hydrophobic interactions.^[^ ^27^ ^]^ The low structural stability of cubosomes prepared using conventional formulations (e.g., MO and PHY) is a key concern for their medical applications.^[^ ^11^, ^28^ ^]^ In contrast, the TA/DMG‐PEG cubosomes prepared herein remained stable in aqueous environment for at least one year at room temperature and 4 °C without changes in size or structure (Figure S6 and Table S2, Supporting Information).

### 2.2 Molecular Insights into the Assembly of PLC‐LNPs

**Figure 2:**
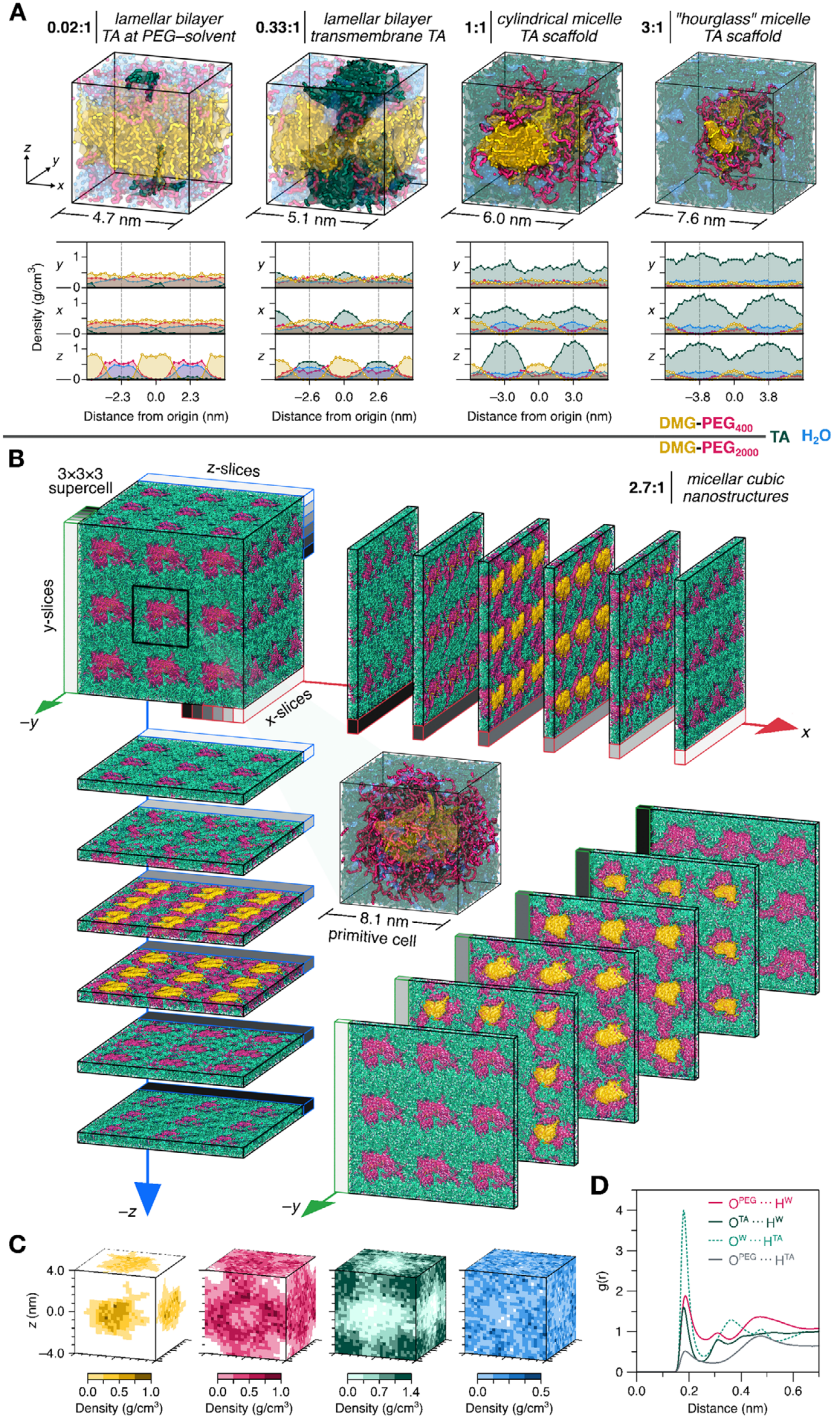
Characterization of solid‐state‐like 3D periodic TA/DMG‐PEG assemblies from MD simulations. A) Atomistic snapshots (top) and molecular density profiles (bottom) of the TA/DMG‐PEG_400_ morphologies show the effects of increasing TA concentration on the structure of the assembled systems. B) Atomistic snapshots illustrating the TA/DMG‐PEG_2000_ (2.7:1) system that experimentally forms micellar cubosomes. The primitive unit cell (center), 3 × 3 × 3 supercell (top left), and 1.5 nm thick molecular slices along Cartesian axes reveal a micellar cubic structure with distinct molecular regions. Lipids aggregate into micelle‐like particles, surrounded by a connected PEG network embedded within a TA scaffold. C) MD‐generated 2D mass density plots of the TA/DMG‐PEG_2000_ (2.7:1) system. D) Radial distribution functions, *g*(*r*), show persistent specific interactions in the assembled TA/DMG‐PEG_2000_ (2.7:1) system structure. Hydrogen bond acceptors: PEG ether (O^PEG^); TA ester/carbonyl, pyranose ether (O^TA^); and water (O^W^). Hydrogen bond donors: TA phenol (H^TA^) and water (H^W^).

MD simulations were employed to understand the role of the polyphenol and lipid components on the formation of the experimentally observed micellar cubosome systems (Figure S7, Supporting Information). All‐atom models were constructed to explore the spontaneous association of TA/lipid mixtures in water and to examine how the resultant supramolecular assemblies are influenced by the i) TA:lipid ratio, ii) amount of hydration, or iii) PEG chain length, as summarized in Figure S7A and Table S3 (Supporting Information). Representative structures with varying concentrations of TA, DMG‐PEG, and water were constructed and simulated to equilibrium. **Figure** **2** and Figures S8–S12 (Supporting Information) show exemplar structures of solid‐state‐like 3D assemblies with different ratios of components of the assemblies. The results indicated that higher TA concentrations led to transition of the lipid mesophase from lamellar bilayers to micelles in DMG‐PEG400 systems (Figure 2A). The concentration profiles of the simulated 3D networks showed that DMG‐PEG provided interfacial boundaries between TA and water (Figure 2A). Figure 2B shows the all‐atom structure of the experimentally consistent network composition (molar ratio between TA and DMG‐PEG was 2.7:1) and highlights the cubic periodic network observed experimentally. The ability of the amphipathic DMG‐PEG to bind to both aqueous and TA media (via hydrophilic region PEG) and form lipid micelles (via hydrophobic region DMG) enabled the formation of these interfacial micellar cubic systems, as evidenced by density profiles (Figure 2C; Figure S13, Supporting Information) and specific interactions between the functional groups of the components (Figure 2D). Similar to the traditional amphiphilic systems, PLC‐LNPs formed by TA and DMG‐PEG were driven by hydrophobic interactions within lipid tails through interfacial energy minimization, evidenced by the simulated all‐atom structure (Figure 2B) and experimental disassembly assays (Figure S5, Supporting Information). The hydrophilic interactions, i.e., hydrogen bonding between PEG, TA, and water, are the major interactions that exist in the hydrophilic region in this micellar cubic structure (Figure 2D). The structure and mobility of all molecular components within the interfacial system in Figure 2B are illustrated in Movie S1A (Supporting Information), and the mobility of the water molecules is shown in Movie S1B (Supporting Information). The water molecules trapped in persistent positions are mostly within the TA scaffold, whereas dynamic water molecules that move within channels are mostly within the PEG region.

### 2.3 Compositional Engineering of PLC‐LNPs

**Figure 3:**
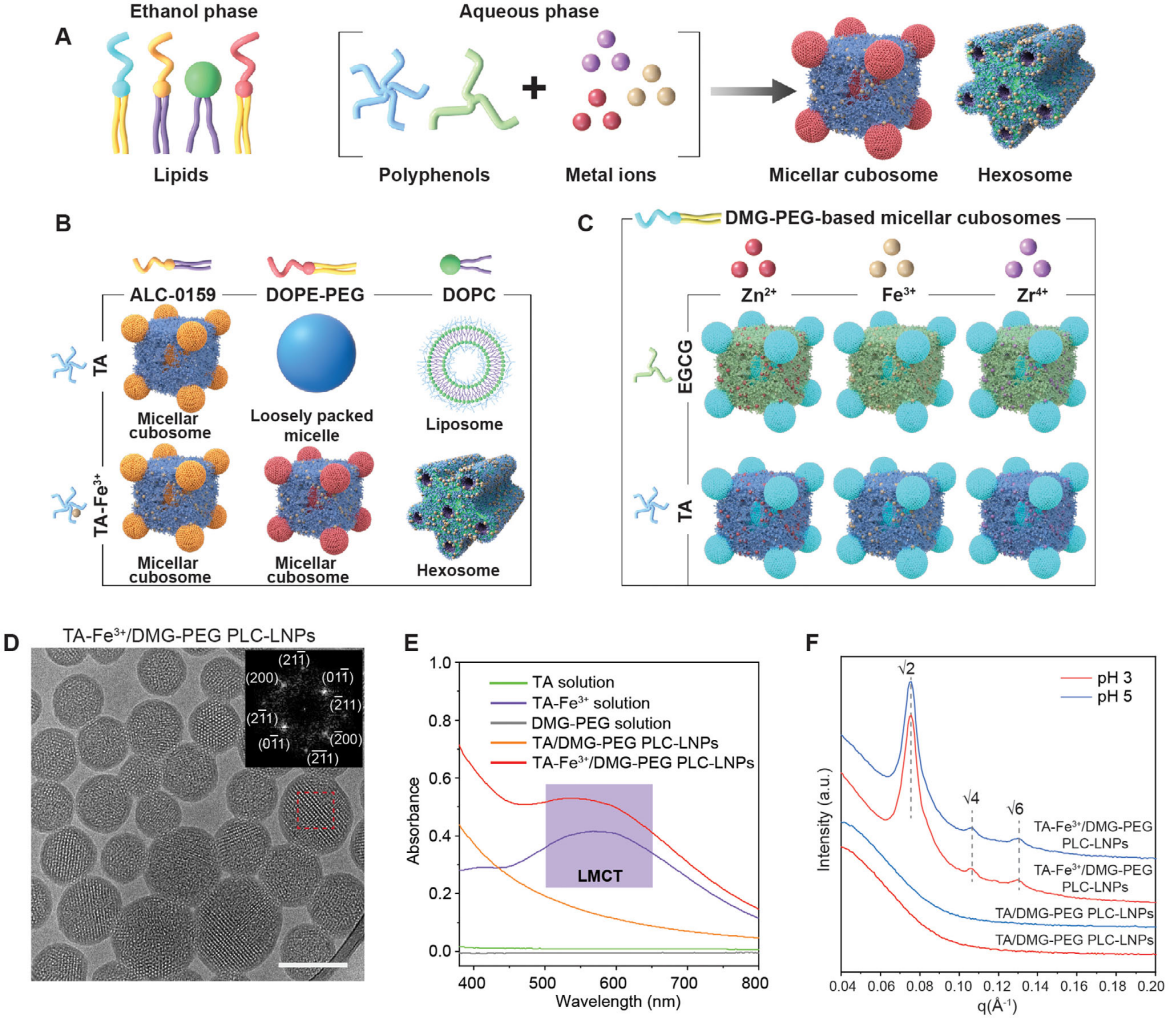
PLC‐LNPs assembled using different building blocks. A) Schematic of the preparation of PLC‐LNPs using different lipids and MPNs. B,C) Library of PLC‐LNPs with different nanostructures assembled from different lipids (B) or MPNs composed of different polyphenols (TA or EGCG) and metal ions (Zn^2+^, Fe^3+^, or Zr^4+^) (C). TA‐Fe^3+^ represents MPN complexes formed from TA and Fe^3+^. D) Cryo‐electron microscopy image of TA‐Fe^3+^/DMG‐PEG PLC‐LNPs. Scale bar is 200 nm. The inset shows a fast Fourier transform image with assigned Miller indices for the section marked with a red frame. E) UV–vis spectra of TA solution, TA‐Fe^3+^ solution, DMG‐PEG solution, and TA/DMG‐PEG and TA‐Fe^3+^/DMG‐PEG PLC‐LNPs. TA‐Fe^3+^/DMG‐PEG PLC‐LNPs featured a ligand‐to‐metal charge transfer (LMCT) band within the range of 450–650 nm, suggesting MPN formation in the PLC‐LNPs. F) 1D SAXS patterns of TA/DMG‐PEG and TA‐Fe^3+^/DMG‐PEG PLC‐LNPs in pH 3 and pH 5 buffers.

The MD simulations revealed that the nanostructures of PLC‐LNPs are influenced by the interactions between polyphenols and lipids, offering the possibility of tuning the nanostructure by altering the assembly building blocks (**Figure** **3**A). Tailoring the nanostructure and composition of lipid–polyphenol assemblies is essential for tuning their biophysical properties, such as membrane curvature and surface topology, which consequently dictate cellular uptake mechanisms and delivery outcomes.^[^ ^29^, ^30^, ^31^, ^32^, ^33^, ^34^ ^]^ Moreover, distinct internal mesophases possess different crystallographic symmetries and diffusion characteristics, enabling the rational modulation of cargo transport and controlled drug release.^[^ ^35^, ^36^, ^37^ ^]^

Changing DMG‐PEG to other types of PEG‐conjugated di‐tetradecyl lipids—i.e., 2‐(polyethylene glycol‐2000)‐*N*,*N*‐ditetradecylacetamide (ALC‐0159) and α‐(3′‐{[1,2‐di(myristyloxy)propanoxy] carbonylamino}propyl)‐ω‐methoxy, polyoxyethylene (PEG‐c‐DMG)—resulted in the formation of *Im3m* micellar cubosomes with different lattice parameters (108 and 122 Å, respectively), after mixing with TA at a TA‐to‐lipid molar ratio of 1.8:1 (Figure 3B; Figure S14A,B and Table S2, Supporting Information). Using a different lipid, i.e., dioleoyl phospholipid (1,2‐dioleoyl‐*sn*‐glycero‐3‐phosphocholine (DOPC)) led to the formation of liposomes with multilamellar nanostructures, after mixing with TA at a TA‐to‐lipid molar ratio of 1.8:1 (Figure 3B; Figure S15C, Supporting Information). This transformation is consistent with a previous study that attributes the multilamellarity of phospholipids to the multiple hydrogen bonding interactions between TA and phosphocholine headgroups, which promote intermembrane adhesion and stacking.^[^ ^38^ ^]^ Mixing TA with PEG‐conjugated dioleoyl phospholipid (1,2‐dioleoyl‐*sn*‐glycero‐3‐phosphoethanolamine‐*N*‐[methoxy(polyethyleneglycol)‐5000)] (DOPE‐PEG)) at the same TA‐to‐lipid ratio resulted in the formation of NPs with loosely packed micellar structures (Figure S14E, Supporting Information). These transitions could be due to the limited capabilities of TA to modulate the interfacial curvature of lipids with long carbon chains.

Incorporating metal ions (Zn^2+^, Fe^3+^, or Zr^4+^) as the third component led to the formation of a metal–phenolic network (MPN) in the PLC‐LNPs (Figure 3E; Figure S16, Supporting Information),^[^ ^39^, ^40^, ^41^ ^]^ which had a negligible influence on the mesophases or lattice parameters of DMG‐PEG‐, ALC‐0159‐, and PEG‐c‐DMG‐based cubosomes (Figure 3B–D; Figures S14A,B, and S17, Supporting Information). However, structural changes from lamellar to inverse hexagonal, and from disordered intermediate phase to Type I micellar cubic were observed in DOPC‐ and DOPE‐PEG‐based PLC‐LNPs, respectively, after incorporating Fe^3+^ into the systems (Figure 3B; Figures S14C and S15 and Table S2, Supporting Information). Although the SAXS peak of the TA–Fe^3^⁺/DOPC NPs is not sufficient for definitive phase identification (Figure S15A, Supporting Information), the NPs display well‐defined lattice fringes characteristic of a hexagonal mesophase, in contrast to the concentric multilamellar morphology observed in TA/DOPC liposomes (Figure S15C,D, Supporting Information). This could be due to the ability of the TA‐Fe^3+^ complex to more effectively modulate the phase change of dioleoyl lipids compared with free TA through the formed MPN in the hydrophilic region. Polyphenols have been shown to form hydrogen bonds with phospholipids and induce lipid bending.^[^ ^42^ ^]^ In addition, metal ions have been reported to coordinate with phospholipids,^[^ ^43^ ^]^ which may promote a more negative curvature in phospholipid‐based assemblies. The differences in phase behavior across lipid types can be qualitatively understood using the CPP value, defined as CPP = *v*/(*a*_0_ × *l*_s_), where *v* is the volume of the hydrophobic tails, *a*_0_ is the effective headgroup area, and *l*_s_ is the tail length.^[^ ^9^, ^36^ ^]^ DMG‐PEG, with short saturated tails and a PEG2000 headgroup, has a small CPP value (<1/3), favoring high‐curvature micellar assemblies.^[^ ^44^ ^]^ Its positive curvature can be reduced through its interactions with TA, thus forming micellar cubosomes. DOPC has a CPP close to 1 and naturally forms lamellar bilayers.^[^ ^10^ ^]^ The observed lamellar‐to‐hexagonal transition between TA/DOPC and TA‐Fe^3+^/DOPC NPs likely reflects an increase in interfacial curvature stress induced by MPN formation, which perturbs the original packing of the lipid bilayer. In contrast, DOPE‐PEG5000, with its large PEG headgroup and long C18 acyl chains, exhibits a very small CPP value and high interfacial constraints imposed by the PEG5000 corona. This unfavorable packing may account for the loosely packed micellar structures in TA/DOPE‐PEG particles, while MPN formation introduces sufficient interfacial modulation to induce an ordered micellar cubic phase in the TA–Fe^3+^/DOPE‐PEG particles by reducing the positive curvature of the DOPE‐PEG micelles.

Although changes in MPN composition, in terms of polyphenols (TA and epigallocatechin gallate (EGCG)), metal ions (Zn^2+^, Fe^3+^, and Zr^4+^), and molar ratios between polyphenols and metal ions (from 200:1 to 1:1) did not influence the nanostructure of DMG‐PEG‐based cubosomes (Figure 3C; Figures S17 and S18 and Table S2, Supporting Information), the incorporation of metal ions increased the stability of the cubosomes under acidic conditions (Figure 3F). This increased stability is possibly due to the counterbalanced effects of reduced MPN coordination and enhanced *π*–*π* interactions of protonated TA (p*K*_a_ ≈ 6) in acidic conditions.^[^ ^39^ ^]^

### 2.4 Encapsulating Cargos Into PLC‐LNPs

**Figure 4:**
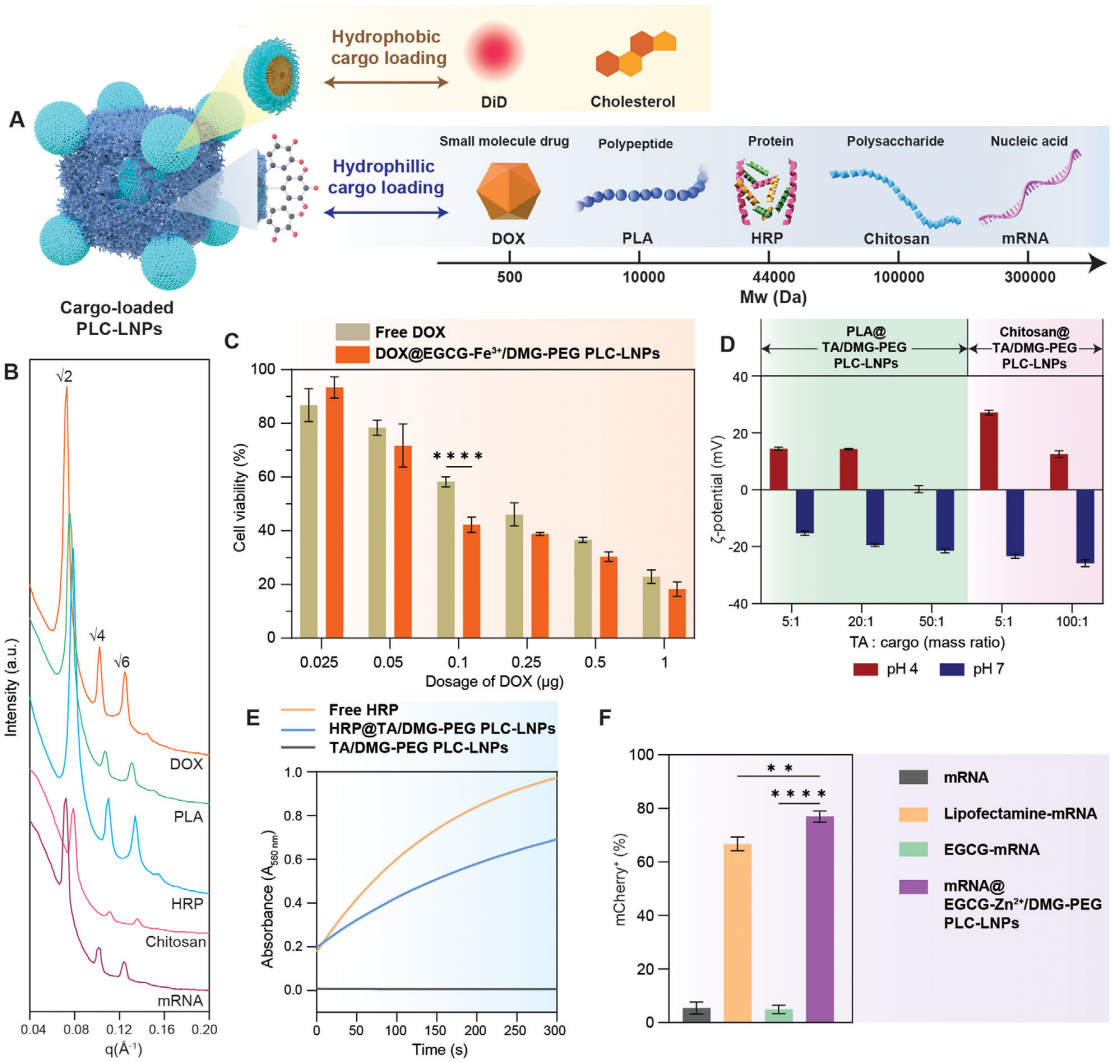
PLC‐LNPs loaded with cargos. A) Schematic of PLC‐LNPs loaded with diverse cargos: hydrophobic cargos including DiD and cholesterol, and hydrophilic cargos including a small‐molecule drug (DOX), a polypeptide (PLA), a protein (HRP), a polysaccharide (chitosan), and a nucleic acid (mRNA). B) 1D SAXS patterns of cargo‐loaded PLC‐LNPs. The peak ratio in all the SAXS patterns is √2:√4:√6. C) Viability of MDA‐MB‐468 cells after incubation with free DOX or DOX@EGCG‐Fe^3+^/DMG‐PEG PLC‐LNPs at different drug dosages. Cell viability (%) was normalized to the cell‐only control group. Data are shown as mean ± SD (*n* = 3 independent replicates) and analyzed by one‐way analysis of variance (ANOVA), ^****^ *p* < 0.0001. D) ζ‐potential of PLA@TA/DMG‐PEG and chitosan@TA/DMG‐PEG PLC‐LNPs assembled using different mass ratios of TA and PLA/chitosan at different pHs. Data are shown as mean ± SD (*n* = 3 independent replicates). E) Time‐dependent absorbance changes upon oxidation of amplex red by different catalytic systems: free HRP, TA/DMG‐PEG PLC‐LNPs, and HRP@TA/DMG‐PEG PLC‐LNPs. F) Percentage of mCherry‐positive (mCherry^+^ (%)) cells in HEK 293T cells treated with mCherry mRNA (mRNA), mCherry mRNA‐lipofectamine MessengerMAX (Lipofectamine‐mRNA), EGCG mixed with mRNA (EGCG‐mRNA), and mCherry mRNA‐loaded PLC‐LNPs (mRNA@EGCG‐Zn^2+^/DMG‐PEG PLC‐LNPs). Data are shown as mean ± SD (*n* = 3 independent replicates) and analyzed by one‐way ANOVA, ^**^ *p* < 0.01 and ^****^ *p* < 0.0001.

LC‐LNPs can encapsulate both hydrophilic and hydrophobic cargos.^[^ ^10^, ^20^ ^]^ To demonstrate their hydrophobic cargo loading capacity, 1,1′‐dioctadecyl‐3,3,3′,3′‐tetramethylindodicarbocyanine perchlorate (DiD) lipid dye and cholesterol were separately loaded into the TA/DMG‐PEG cubosomes (**Figure** **4**A; Figures S19 and S20, Supporting Information). The DiD loading efficiency of TA/DOPC liposomes and TA/DMG‐PEG cubosomes was compared to evaluate the functional advantages of cubic mesophases. As observed from Figure S21 (Supporting Information), TA/DOPC liposomes showed limited DiD encapsulation efficiency (≈10%) at a high DiD dose (2%), whereas TA/DMG‐PEG cubosomes maintained high encapsulation efficiencies (>95%) of DiD at both high and low DiD mole percentages (0.2% and 2%), suggesting enhanced hydrophobic cargo loading capacity of the cubosome system. Intracellular colocalization analysis on those DiD‐labeled NPs further revealed that the TA/DMG‐PEG cubosomes displayed higher endosomal escape efficiency in MDA‐MB‐231 cells, compared to the TA/DOPC liposomes (Figures S22 and S23, Supporting Information), which could be attributed to the distinct structural features of the cubic mesophase. Incorporating 10% cholesterol into the lipid phase increased the lattice parameter of the cubosomes from 117.3 to 122.3 Å (Table S2, Supporting Information), indicating cholesterol intercalation within the lipid matrix. The cubic phase became less ordered, and the lattice parameter increased to 134.7 Å when the percentage of cholesterol increased to 30% (Table S2, Supporting Information), likely due to excess cholesterol perturbing the fine curvature balance and packing geometry necessary to maintain the *Im3m* symmetry.

Polyphenols display multiple interactions with diverse molecules through coordination, hydrogen bonding, *π* interactions, hydrophobic interactions, and electrostatic interactions.^[^ ^45^, ^46^, ^47^ ^]^ Accordingly, we also evaluated the ability of the PLC‐LNPs for hydrophilic cargo loading into the hydrophilic regions in the cubosome matrix (Figure 4A). The small‐molecule anticancer drug DOX was loaded into the EGCG‐Fe^3+^/DMG‐PEG cubosomes with up to 94% loading efficiency via a post‐loading strategy (Figures S24–S26, Supporting Information). The high DOX loading is attributed to the presence of continuous hydrophilic regions within the cubosome matrix, as well as the affinity of DOX for both polyphenols and metal ions mediated through hydrophobic interactions and coordination, respectively.^[^ ^48^, ^49^, ^50^ ^]^ The cubosomes showed minimal size changes after postloading of DOX (Figure S27, Supporting Information). Furthermore, the DOX‐loaded EGCG‐Fe^3+^/DMG‐PEG cubosomes (DOX@EGCG‐Fe^3+^/DMG‐PEG cubosomes) showed comparable cytotoxicity with free DOX to breast cancer cells (MDA‐MB‐468) (Figure 4C) and MDA‐MB‐231 cells (Figure S28, Supporting Information), whereas the cubosomes without DOX loading exhibited negligible cytotoxicity (Figure S29, Supporting Information). Diverse types of biomolecules—including polypeptides (e.g., poly‐l‐arginine (PLA)), proteins (e.g., horseradish peroxidase (HRP)), polysaccharides (e.g., chitosan), nucleic acids (e.g., mCherry mRNA)—have been reported to exhibit distinct interactions with polyphenols^[^ ^27^, ^51^, ^52^, ^53^ ^]^ and were successfully incorporated into the cubosomes without disrupting the cubic nanostructure (Figure 4B). Compared to TA/DOPC liposomes, TA/DMG‐PEG cubosomes exhibited a higher PLA encapsulation efficiency (Figure S21, Supporting Information), likely due to their distinct internal nanostructure that more efficiently accommodates macromolecular cargos. Varying the ratio between TA and PLA or chitosan influenced the cargo loading efficiency (Figures S30 and S31, Supporting Information), as well as the size of the assembled cubosomes, which was tunable and ranged between 146 and 423 nm (Figures S32 and S33, Supporting Information). Furthermore, the PLA‐ and chitosan‐loaded TA‐based cubosomes (PLA@TA/DMG‐PEG and chitosan@TA/DMG‐PEG cubosomes) exhibited surface charge reversal properties, changing from negative or neutral (at pH 7) to positive (at pH 4) (Figure 4D). The TA/DMG‐PEG cubosomes also enabled HRP loading with a negligible size change (Figures S34–S36, Supporting Information). The HRP‐loaded TA‐based cubosomes (HRP@TA/DMG‐PEG cubosomes) showed comparable catalytic activity to free HRP in the oxidation of amplex red in the presence of H_2_O_2_ (Figure 4E). Postloading mRNA into cubosomes was achieved by incubating the EGCG‐Zn^2+^/DMG‐PEG cubosomes with mRNA dispersed in EGCG solution. This formulation was selected because Zn^2^⁺ can coordinate with the phosphate backbone of mRNA,^[^ ^54^ ^]^ which is expected to enhance mRNA loading efficiency. Moreover, the addition of EGCG during postloading enhanced the mRNA loading efficiency (Figure S37, Supporting Information), probably due to the interactions between EGCG and the phosphate backbone of mRNA via hydrogen bonds.^[^ ^55^ ^]^ The cubosomes showed negligible size change after mRNA loading (Figure S38, Supporting Information), and the mRNA‐loaded cubosomes (mRNA@EGCG‐Zn^2+^/DMG‐PEG cubosomes) showed ≈75% transfection efficiency with negligible cytotoxicity (Figure 4F; Figures S39–S41, Supporting Information). mRNA was more rapidly released (≈80% within 3 h) from cubosomes in a cytosol‐mimicking reductive buffer (10 mm glutathione (GSH)) than in water (<10% over 24 h) or cell culture medium (≈30% at 24 h) (Figure S42, Supporting Information). This GSH‐responsive behavior is attributed to the high affinity of GSH for both Zn^2+^ and EGCG, through coordination^[^ ^56^ ^]^ and noncovalent interactions,^[^ ^57^ ^]^ respectively, leading to partial disassembly of the cubosome matrix.

## 3 Conclusion

The present findings reveal that lipid mesophases can be modulated by naturally occurring polyphenols, and the polyphenol/lipid system can assemble into NPs with ordered *Im3m* micellar cubic nanostructures in which lipid micelles are closed‐packed by the polyphenol scaffold. The nanostructures of this system are governed by the interactions between polyphenols and lipids and can be tuned by altering the assembly building blocks. The polyphenol/lipid nanostructured system allows the loading of a range of cargos for diverse potential applications owing to the adherent properties of polyphenols and the presence of hydrophobic lipid cores. The tuning of the phase behavior and physicochemical properties of nanostructured NPs is commonly achieved through the synthesis of new amphiphiles or the interplay between different amphiphiles. In comparison, our work demonstrates that the formation of ordered nanostructures can also be achieved through the interplay between amphiphiles and polyphenols. Furthermore, the physicochemical properties (e.g., pH stability) of the nanostructured NPs can be tuned by incorporating different guest molecules. This work provides a platform for the design and assembly of nanostructured and functional LNPs for diverse applications.

## Conflict of Interest

An international patent application (PCT/AU2025/050116; WO2025/171440) covering compositions, methods, and uses for mRNA delivery was filed by The University of Melbourne. F.C. is a shareholder of Messenger Bio Pty. Ltd. All other authors declare that they have no competing interests.

## Supporting information



Supporting Information

Supplementary Movie

## Data Availability

The data that support the findings of this study are available from the corresponding author upon reasonable request.
