# Supplementary material for: Polyphenol‐Mediated Engineering of Lipid Nanoparticles With Crystalline Mesophases
Source: Adv Mater. 2025 Sep 15;38(6):e05830. doi: 10.1002/adma.202505830 (PMC12848655; doi:10.1002/adma.202505830)
Supplement: Supplementary file 1 — Supporting Information [file ADMA-38-e05830-s001.pdf]

ADVANCED MATERIALS

Supporting Information

for *Adv. Mater.*, DOI 10.1002/adma.202505830

Polyphenol-Mediated Engineering of Lipid Nanoparticles With Crystalline Mesophases

Shiyao Li, Patrick Charchar, Haitao Yu, Zhixing Lin, Hamish G. Brown, Wanjun Xu, Chan-Jin Kim, Irene Yarovsky, Yi Ju and Frank Caruso**

Supporting Information

Polyphenol-Mediated Engineering of Lipid Nanoparticles with Crystalline Mesophases

Shiyao Li, Patrick Charchar, Haitao Yu, Zhixing Lin, Hamish G. Brown, Wanjun Xu, Chan-Jin Kim, Irene Yarovsky, Yi Ju, and Frank Caruso**

*Corresponding authors. Email: david.ju@onjcri.org.au; fcaruso@unimelb.edu.au

Materials

Tannic acid (TA, ACS reagent), epigallocatechin gallate (EGCG, ≥95%), urea (99.0–100.5%), sodium chloride (NaCl, ≥99.5%), Tween-20 (BioXtra, viscous liquid), iron(III) chloride hexahydrate ($FeCl_3·6H_2O$, ≥98.0%), zinc(II) chloride ($ZnCl_2$, ≥98.0%), zirconium(IV) chloride ($ZrCl_4$, ≥99.5%), glycine (≥99.0%), 3-(*N*-morpholino)propanesulfonic acid (MOPS, ≥99.5%), hydrochloric acid (HCl, 36.5–38.0%), sodium hydroxide (NaOH, ≥98%), sodium acetate (≥99%), acetic acid, sodium phosphate dibasic heptahydrate, sodium phosphate monobasic monohydrate, dimethyl sulfoxide-d6 (DMSO-d6), 1,2-dioleoyl-*sn*-glycero-3-phosphoethanolamine-*N*-[methoxy(polyethylene glycol)-5000] (ammonium salt) (DOPE-PEG), Dulbecco's phosphate-buffered saline (DPBS), nitric acid (HNO_3) (65%), cholesterol (≥99%), doxorubicin hydrochloride (DOX), poly-L-arginine (PLA), horseradish peroxidase (HRP), chitosan (low molecular weight), triethylamine (≥99.5%), dimethyl sulfoxide (DMSO), and L-glutathione reduced (GSH) (≥98.0%) were purchased from Sigma-Aldrich. Slide-A-Lyzer MINI Dialysis Devices (20K molecular weight cutoff), 1,1′-dioctadecyl-3,3,3′,3′-tetramethylindodicarbocyanine perchlorate (DiD), LysoTracker Green DND-26, Hoechst 33342, fluorescein-5-isothiocyanate (FITC) and Opti-MEM were purchased from Thermo Fisher Scientific. 1,2-Dimyristoyl-*rac*-glycero-3-methoxypolyethylene glycol-2000 (DMG-PEG) and 1,2-dioleoyl-*sn*-glycero-3-phosphocholine (DOPC) were purchased from Avanti Polar Lipids. 2-[(Polyethylene glycol)-2000]-*N*,*N*-ditetradecylacetamide (ALC-0159) and α-(3′-{[1,2-di(myristyloxy)propanoxy] carbonylamino}propyl)-ω-methoxy, polyoxyethylene (PEG-c-DMG) were purchased from MedChemExpress. Lipofectamine MessengerMAX, Dulbecco's modified Eagle medium (DMEM), fetal bovine serum (FBS), and 2,3-bis[2-methoxy-4-nitro-5-sulfophenyl]-2*H*-tetrazolium-5-carboxyanilide inner salt (XTT) were purchased from Life Technologies. mCherry mRNA and cyanine5 (cy5)-labeled green fluorescent protein (GFP) mRNA were purchased from Messenger Bio. MDA-MB-231, MDA-MB-468, and HEK293T cells were purchased from the American Type Culture Collection (USA). High-purity water with a resistivity of 18.2 MΩ cm was obtained from an inline Millipore RiOs/Origin water purification system. All solutions were freshly prepared for immediate use in each experiment.

Methods

General characterization

(1) Size measurements of the nanoparticles (NPs) were performed on a Zetasizer Nano-ZS (Malvern Instruments, UK) instrument. ζ-Potential measurements of the NPs were performed at pH 7.4 in phosphate buffer (5 mM) on a Zetasizer Nano-ZS.

(2) NP concentrations were measured on a Malvern NanoSight NS300 instrument fitted with a 405 nm laser (65 mW output).

(3) Nuclear magnetic resonance (NMR) spectroscopy was conducted on a Varian Unity 400 MHz spectrometer at room temperature. Samples were dissolved in DMSO-d6 (0.65 mL).

(4) UV–visible absorption measurements were performed on an Analytik Jena SPECORD 250 PL instrument.

(5) Super-resolution (via lattice-structured illumination microscopy (lattice-SIM)) microscopy imaging was conducted on a lattice-structured illumination microscope (Zeiss Elyra 7).

(6) Fluorescence intensity measurements were conducted on a fluorescence spectrophotometer (FS5, Edinburgh Instruments).

Preparation of polyphenol-based nonlamellar liquid crystalline lipid NPs (PLC-LNPs)

The LC-LNPs were formulated using a NanoAssemblr microfluidic device (Ignite System, Precision NanoSystems, Canada). The total flow rate ratio was set as 12 mL min^{-1} and the flow rate ratio of the aqueous phase to ethanol phase was set as 4:1 unless specified.

To prepare DMG-PEG NPs, DMG-PEG in ethanol was mixed with Milli-Q water in aqueous phase. To prepare TA/DMG-PEG NPs, DMG-PEG in ethanol (2 mM) was mixed with a TA aqueous solution to achieve varying molar ratios between TA and DMG-PEG from 0.33:1 to 10:1. Cubosomes were specifically obtained at TA:DMG-PEG molar ratios equal to or higher than 1:1. TA/DMG-PEG cubosomes were also formulated using a higher DMG-PEG concentration of 10 mM, and the molar ratio between TA and DMG-PEG was adjusted from 1:1 to 3:1.

To prepare TA-based LC-LNPs using different lipids, ALC-0159, PEG-c-DMG, or DOPE-PEG (2 mM in ethanol) was mixed with TA (1.5 mg mL^{-1} in water), and DOPC (1 mg mL^{-1} in ethanol) was mixed with TA (1 mg mL^{-1} in water).

To prepare metal–phenolic network (MPN)/DMG-PEG LC-LNPs, an MPN aqueous solution was first prepared by mixing polyphenols (TA or EGCG; 1.5 mg mL^{-1}) and metal ions ($ZnCl_2$, $FeCl_3·6H_2O$, or $ZrCl_4$; 1 mg mL^{-1}) in aqueous phase to yield known polyphenol-to-metal ions molar ratios of 200:1 to 1:1 (for TA-Zn^{2+} and TA-Fe^{3+} MPN), 200:1 to 2:1 (for TA-Zr^{4+} MPN), and 200:1 to 10:1 (for EGCG-Zn^{2+}, EGCG-Fe^{3+}, and EGCG-Zr^{4+}). Then, the MPN aqueous solution was mixed with DMG-PEG (2 mM) in ethanol. The flow rate ratio of the aqueous phase to ethanol phase was changed to 5:1 for EGCG-based LC-LNPs.

To prepare TA-Fe^{3+}-based LC-LNPs using different lipids, 1.5 mg mL^{-1} TA was first mixed with $FeCl_3·6H_2O$ at a TA-to-Fe^{3+} molar ratio of 1:1, and then TA-Fe^{3+} MPN was mixed with ALC-0159, PEG-c-DMG, DOPE-PEG (2 mM, in ethanol), or DOPC (1 mg mL^{-1}, in ethanol).

The PLC-LNPs were dialyzed against Milli-Q water using dialysis membrane (Slide-A-Lyzer MINI Dialysis Devices, 20K molecular weight cutoff) at room temperature for at least 2 days. The Milli-Q water was replaced at least every 24 h.

Preparation of cargo-loaded PLC-LNPs

To prepare DiD@TA/DMG-PEG cubosomes, DiD (0.2 mol%) was first added to the DMG-PEG lipid phase (2 mM in ethanol). This lipid solution was then mixed with TA using the NanoAssemblr microfluidic device at a TA-to-DMG-PEG molar ratio of 3:1. The total flow rate was set to 12 mL min^{-1}, with an aqueous-to-ethanol phase flow rate ratio of 3:1.

To prepare cholesterol@TA/DMG-PEG cubosomes, cholesterol (10 and 30 mol%) was first added to the DMG-PEG lipid phase (2 mM in ethanol). This lipid solution was then mixed with TA using the NanoAssemblr microfluidic device at a TA-to-DMG-PEG molar ratio of 3:1. The total flow rate was set to 12 mL min^{-1}, with an aqueous-to-ethanol phase flow rate ratio of 3:1.

To prepare DOX@EGCG-Fe^{3+}/DMG-PEG cubosomes, 5×10^7 µL^{-1} EGCG-Fe^{3+}/DMG-PEG cubosomes (400 µL, EGCG-to-Fe^{3+} molar ratio was 10:1) were incubated with DOX aqueous solution (20 µL, 1 µg µL^{-1}) in an Eppendorf thermomixer at 4 °C and 600 rpm for 2 h. The DOX-loaded cubosomes were first washed with Milli-Q water by centrifugation at 1000 *g* for 5 min. The resulting supernatant was then subjected to sequential centrifugation at 2000 *g* and 3000 *g* for 5 min each to ensure complete particle removal (<1% residual particles), as confirmed by nanoparticle tracking analysis. This supernatant was used to quantify DOX loading efficiency. Excessive centrifugal force and prolonged spinning were deliberately avoided to prevent irreversible cubosome aggregation. The purified pellet was subsequently redispersed in Milli-Q water for cell viability experiments.

To prepare PLA@TA/DMG-PEG cubosomes, TA (1.5 mg mL^{-1} in Milli-Q water) was mixed with PLA (1 mg mL^{-1} in Milli-Q water) at TA-to-PLA mass ratios of 50:1, 20:1, and

5:1. Then, the TA–PLA suspension was mixed with DMG-PEG (2 mM in ethanol) in the NanoAssemblr microfluidic device. The total flow rate ratio was set as 12 mL min^{-1}, and the flow rate ratio of aqueous phase to ethanol phase was set as 4:1.

To prepare chitosan@TA/DMG-PEG cubosomes, TA (1.5 mg mL^{-1} in Milli-Q water) was mixed with chitosan (1 mg mL^{-1} in 1% acetic acid) at TA-to-chitosan mass ratios of 100:1, 50:1, 20:1, and 5:1. Then, the TA-chitosan suspension was mixed with DMG-PEG (2 mM in ethanol) in the NanoAssemblr microfluidic device. The total flow rate ratio was set as 12 mL min^{-1} and the flow rate ratio of the aqueous phase to ethanol phase was set as 4:1.

To prepare HRP@TA/DMG-PEG cubosomes, 5×10^7 μL^{-1} TA/DMG-PEG cubosomes (1 mL) (1:1 TA:DMG-PEG, 10 mM DMG-PEG) were incubated with HRP aqueous solution (50 μL, 1 μg μL^{-1}) in an Eppendorf thermomixer at 4 °C and 600 rpm for 1 h. The HRP-loaded cubosomes were then purified by iterative low-speed centrifugation. The sample was centrifuged at 1000 *g* for 3 min, and the supernatant was carefully collected and subjected to repeated centrifugation under the same conditions until it became clear. Excessively high centrifugal force or prolonged centrifugation was avoided to prevent irreversible pellet aggregation. The supernatant was collected for measurement of HRP loading efficiency via UV–vis spectroscopy. Note, the microBCA assay is incompatible with our particle system (Fig. S32). The purified HRP@TA/DMG-PEG cubosomes were used for catalysis experiments.

mRNA@EGCG-Zn^{2+}/DMG-PEG cubosomes were prepared by incubating 2×10^8 μL^{-1} EGCG-Zn^{2+}/DMG-PEG cubosomes (100 μL, molar ratio between EGCG and Zn^{2+} was 10:1) with mCherry mRNA (4 μL, 1 μg μL^{-1}) dispersed in EGCG (100 μL, 3 mg mL^{-1}) in an Eppendorf thermomixer at 4 °C and 600 rpm for 3 h. The sample was centrifuged at 2000 *g* for 3 min and subjected to repeated centrifugation under the same conditions until it became clear. Excessively high centrifugal force or prolonged centrifugation was avoided to prevent irreversible pellet aggregation. The purified mRNA@EGCG-Zn^{2+}/DMG-PEG cubosomes were used for transfection experiments. To assess mRNA loading efficiency, cy5-labeled GFP mRNA was used instead of mCherry mRNA. The mRNA-loaded cubosomes were prepared and purified as above, and the supernatant was carefully collected. The fluorescence intensity of cy5 in the supernatant was measured using a microplate reader (TECAN, InfiniteM200, Switzerland) (excitation: 646 nm, emission: 662 nm), and the loading efficiency was calculated based on the standard curve. Note that the RiboGreen assay was not used for mRNA quantification, as our particle formulation contains phenolic, salt, and PEG components that are incompatible with the assay reagents, according to manufacturer's guidance (Thermo Fisher Scientific, R11490).

Stability of PLC-LNPs

To evaluate the stability of the PLC-LNPs under different conditions, the NPs were incubated in glycine–HCl (20 mM, pH 3.0), acetate acid buffer (20 mM, pH 5.0), phosphate buffer (20 mM, pH 7.0), MOPS buffer (20 mM, pH 9.0), or Milli-Q water for the desired times. To determine the possible driving forces for the assembly of PLC-LNPs, the NPs were incubated in urea (50 mM), Tween 20 (50 mM), or NaCl (10 mM) for 24 h. Changes in size were measured on a Zetasizer Nano-ZS instrument. Data are shown as the mean ± standard deviation (SD) of three independent measurements.

Synchrotron small-angle X-ray scattering (SAXS)

Synchrotron SAXS experiments were conducted at the SAXS/WAXS beamline at the Australian Synchrotron, ANSTO. A wavelength of k = 1.128 Å (11.0 keV) and a camera length of 1.6 m were employed. Samples were first loaded into a UV-clear 96 well plate (Greiner Bio-One, Germany) and screened with an exposure time of 2 s. A Dectris-Pilatus 1 M detector was used to record the 2D X-ray diffraction images, which were subsequently integrated into 1D diffraction plots using the ScatterBrain (V 1.230) software. The scattering vector, q, is defined

as $q = 4\pi\sin\theta/\lambda$ where θ is the scattering angle and λ is the wavelength. Subsequently, d spacing can be calculated using Bragg's law, $d = 2\pi/q$. The lattice parameters of the mesophases can be calculated, as follows, where (h,k,l) are Miller indices assigned to the mesophase:

$$\text{Lattice parameter} = d(h^2 + k^2 + l^2)^{1/2} \text{ for cubic phase}$$

$$\text{Lattice parameter} = (2/\sqrt{3}) \times d(h^2 + k^2 + hk)^{1/2} \text{ for hexagonal phase}$$

Cryo-transmission electron microscopy

A Thermo Fisher Scientific Vitrobot Mk IV was used to prepare the NP samples for cryo-transmission electron microscopy. The samples were pipetted in aliquots (4 µL) onto Ted Pella lacey carbon 400 mesh grids, glow discharged at 15 mA for 30 s in a Quorum GloQube glow discharger, and plunge-frozen in liquid ethane. The Vitrobot settings were set at a chamber humidity of 95% and temperature of 22 °C, and a blot force of 1 and blot time of 4 s. Imaging was conducted on a Thermo Fisher Scientific Tecnai F30 transmission electron microscope operated at 200 kV with a Gatan 626 cryo-transfer specimen holder and a defocus level of −1 µm.

Metal content quantification in TA-metal ion/DMG-PEG cubosomes

The metal content in the TA-metal ion/DMG-PEG cubosome samples was quantified using inductively coupled plasma-optical emission spectroscopy (ICP-OES). HNO_3 solution (1 mL, 65%) was added to an aliquot (2 mL) of a purified cubosome dispersion and incubated for 1 h at room temperature (22 °C) to disassemble the cubosomes and release the coordinated metal ions. The resulting solution was diluted with ultrapure water to 5% HNO_3 and filtered through a 0.22 µm polytetrafluoroethylene membrane filter (Millex) to remove aggregates. Samples were analyzed using a Varian 720-ES ICP-OES system. Standard solutions of Zn, Fe, and Zr were used to generate external calibration curves for quantification. The loading efficiency of metal ions was calculated as the percentage of metal mass detected in the purified cubosomes relative to the total metal added during formulation.

Comparison of cargo loading efficiency of TA/DOPC liposomes and TA/DMG-PEG cubosomes

To prepare PLA-loaded TA/DOPC liposomes and TA/DMG-PEG cubosomes, TA (1.5 mg mL^{-1} in Milli-Q water) was first mixed with FITC-labeled PLA at a TA-to-PLA mass ratio of 50:1. The TA–PLA mixture was then mixed with DOPC or DMG-PEG (1.25 mM in ethanol) in the NanoAssemblr microfluidic device. To prepare DiD-loaded TA/DOPC liposomes and TA/DMG-PEG cubosomes, lipids (DOPC or DMG-PEG, 1.25 mM in ethanol) were first mixed with DiD with a DiD mole percentage of 0.2% and 2%. The lipid mixture was then mixed with TA (1.5 mg mL^{-1} in Milli-Q water) in the NanoAssemblr microfluidic device. The total flow rate ratio was set as 12 mL min^{-1}, and the flow rate ratio of the aqueous phase to ethanol phase was set as 3:1. After formulation, the NPs were centrifuged at 15,000 g for 10 min, and the supernatant was collected to determine the amount of unencapsulated cargo. The encapsulation efficiency was calculated using the following formula:

$$\text{Encapsulation efficiency (\%)} = \left(1 - \frac{C_{\text{free}}}{C_{\text{total}}}\right) \times 100\%$$

where C_{free} is the concentration of unencapsulated PLA or DiD in the supernatant and C_{total} is the total amount added initially. The fluorescence intensity of FITC (excitation/emission wavelengths = 495/519 nm) and DiD (excitation/emission wavelengths = 644/665 nm) was measured using a microplate reader to determine cargo concentrations based on standard calibration curves.

Intracellular colocalization experiments

MDA-MB-231 cells were seeded in the 8-well Lab-Tek-Chamber slide at a density of 40,000 cells per well in DMEM supplied with 10% FBS (400 μL) for 18 h. After incubation, the culture media was removed and replaced with fresh media (200 μL) containing 8 × 10^7 DiD (0.2 mole%)-labeled NPs, followed by 4 h incubation. The media was then removed, and the cells were gently washed twice with DPBS to remove excess NPs. LysoTracker Green DND-26 (50 nM in cell media) was added to the cells and incubated for 1 h. The cells were gently washed three times with DPBS and incubated with Hoechst 33342 (1 μg mL^{-1} in DPBS) for 10 min to stain the nucleus. Finally, the cells were live-imaged using a Nikon A1R confocal microscope with a 40× water immersion objective. Pearson correlation coefficients (PCCs) and color scatter plots were obtained using the colocalization analysis in WCIF ImageJ software.

Evaluation of cell viability by XTT assay

XTT assays were performed on MDA-MB-231 and MDA-MB-468 cells with passages less than 40 to assess the cell toxicity of free DOX, and EGCG-Fe^{3+}/DMG-PEG cubosomes before and after loading with DOX. The cells were seeded on a 96-well plate (Costar, Corning, USA) at a density of 10,000 cells per well in DMEM supplied with 10% FBS (100 μL) overnight. Then, the culture media was removed and replaced with fresh media containing free DOX or DOX@EGCG-$Fe^{3+/}$DMG-PEG cubosomes with DOX dosages of 0.025, 0.05, 0.1, 0.25, 0.5, and 1 μg per well. The same amounts of EGCG-Fe^{3+}/DMG-PEG cubosomes without DOX loading were added to the cells as a negative control. The cells were further incubated for 24 h.

XTT assays were performed on HEK 293T cells with passages less than 20 to assess the cell toxicity of mRNA@EGCG-Zn^{2+}/DMG-PEG cubosomes. The cells were seeded on a 96-well plate (Costar, Corning, USA) at a density of 10,000 cells per well in DMEM supplied with 10% FBS (100 μL) overnight. Then, the culture media was removed and replaced with Opti-MEM media (for cell control) or Opti-MEM media containing the mRNA@EGCG-Zn^{2+}/DMG-PEG cubosomes at final mRNA concentrations of 0.2, 0.5, 1, 1.5, and 2 μg mL^{-1} for 6 h. After the treatment, the media was replaced with fresh media (DMEM supplied with 10% FBS) and the cells were further incubated for 18 h.

After incubation, fresh media containing activated XTT (9 mL of 0.2 mg mL^{-1} XTT in complete DMEM media was activated by adding 22.5 μL of 0.6 mg mL^{-1} *N*-methyl dibenzopyrazine methyl sulfate in DPBS) and cells were incubated for 3 h. Finally, cells were screened on a microplate reader. Absorbance readings were measured at 475 nm and a reference wavelength of 675 nm was used. Cell viability was expressed as a percentage by normalizing the absorbance to untreated cells. All experiments were performed in triplicates, and data are presented as mean ± SD.

FITC labeling of PLA and chitosan

FITC labeling of PLA was performed following a previously reported method[1] with minor modifications. Briefly, PLA (40 mg) and triethylamine (6 μL, 20 mol% amine basis) were dissolved in anhydrous DMSO (600 μL) under N_2 and stirred at room temperature for 30 min. FITC (8 mg, 10 mol% amine) was dissolved in anhydrous DMSO (600 μL) and added dropwise to the above mixture, followed by stirring under N_2 in the dark at room temperature (22 °C) for 20 h. To quench unreacted amines, 1 M HCl (2 drops) was added to the reaction mixture. The mixture was then dialyzed against methanol for 24 h and then against water over 48 h in the dark with daily water replacement, followed by freeze-drying under vacuum overnight.

FITC-labeled chitosan was synthesized by adapting previously reported protocols.[2–4] Briefly, FITC (5 mg) was dissolved in anhydrous methanol (7.5 mL) and added to 1% (w/v)

chitosan solution in 0.1 M acetic acid (5 mL). The mixture was stirred at room temperature (22 °C) in the dark for 20 h. The pH was then adjusted to ~8.5 using 0.5 M NaOH to precipitate FITC-labeled chitosan. The precipitate was collected by centrifugation (18,000 *g*, 10 min), and washed repeatedly with water until the supernatant was non-fluorescent. The pellet was redissolved in 0.1 M acetic acid (4 mL) and dialyzed against Milli-Q water (250 mL) for 3 days with daily water replacement. The dialyzed solution was freeze-dried under vacuum overnight.

Catalytic activity studies

The catalytic activity of HRP-loaded cubosomes was evaluated by the H_2O_2–amplex red colorimetric reaction. Briefly, free HRP, cubosomes, or HRP@TA/DMG-PEG cubosomes were mixed with MOPS solution (10 mM, pH 7.4) containing H_2O_2 (20 mM) and amplex red (1 mg mL^{-1}). Changes in the absorbance of the red oxidation product (resorufin) at 560 nm were monitored by UV–vis spectroscopy. The kinetic behavior of HRP was studied by monitoring the absorbance at 560 nm at 10 s intervals by UV–vis spectroscopy.

mRNA transfection study

HEK 293T cells with passages less than 20 were seeded on a 48-well plate at a density of 80,000 cells per well in DMEM supplied with 10% FBS (0.5 mL) overnight. Cells were incubated with mCherry mRNA (mRNA), mCherry mRNA−lipofectamine construct (Lipofectamine-mRNA), EGCG-mRNA mixture (EGCG-mRNA), or mRNA-loaded cubosomes (mRNA@EGCG-Zn^{2+}/DMG-PEG cubosomes) in Opti-MEM media for 6 h, with a final mRNA concentration of 2 µg mL^{-1}. The medium was removed and replaced with complete DMEM medium. After 18 h, the culture media was removed and cells were washed twice with PBS and detached using trypsin. The cells expressing mCherry were analyzed by flow cytometry (LSRFortessa, BD Bioscience).

mRNA release study

cy5-mRNA-loaded cubosomes were incubated in ultrapure water, Opti-MEM cell culture media, or 10 mM GSH in 10 mM phosphate buffer (pH 7.4) at 37 °C. At each timepoint, the cubosomes were centrifuged at 6000 *g* for 5 min for complete removal of cubosomes. The supernatant was collected for fluorescence measurements using a plate reader. The amount of released cy5-mRNA was quantified by comparing the fluorescence intensity to that of the total mRNA encapsulated in the stock (pre-incubation) sample.

Statistical Analysis

All data are reported as mean ± standard deviation (SD), with the sample size (n) provided in the respective figure legends. For comparisons among three or more groups, statistical significance was evaluated using one-way analysis of variance followed by Tukey's post hoc test. A p-value of less than 0.05 was considered statistically significant (*$p < 0.05$, **$p < 0.01$, ***$p < 0.001$, ****$p < 0.0001$). All statistical analyses were performed using GraphPad Prism 10.4.0 (GraphPad Software, San Diego, CA, USA). Graphical presentations were performed using GraphPad Prism 10.4.0 and OriginPro 2024 (OriginLab, Northampton, MA, USA).

Minimum information reporting in bio–nano experimental literature (MIRIBEL)

The studies conducted herein, including material characterization, biological characterization, and experimental details, conform to the MIRIBEL reporting standard for bio–nano research,[5] and we included a companion checklist of these components therein.

Supplementary Text

Molecular dynamics (MD): model information

All-atom models were constructed to explore the spontaneous association of TA/lipid mixtures in water and to examine how the resultant supramolecular assemblies are influenced by three factors, as summarized in Fig. S7A: (i) the ratio of TA to lipid, (ii) the amount of hydration, and (iii) the length of the PEG chains. Each simulation contained a fixed number of particles with a specific TA/lipid composition (Table S3). The amount of water in the system was used to emulate a particular timepoint during the mesoscale self-assembly process. To represent the final "dry" structures formed experimentally, models were created under low hydration conditions that contain 15 water molecules per TA/lipid molecule (93.8 mol% water). This molar fraction of water is an approximate estimate of the amount of water in the 2:1 TA:DMG-PEG (2 mM) cubic assembly (Table S2) that has a final experimental TA-to-DMG-PEG molar ratio of 2.7:1 (Fig. S3). Systems modelled with 150 water molecules per TA/lipid molecule (99.3 mol% water) were used to represent moderately hydrated transient structures observed in the intermediary stages of assembly. Full hydration scenarios, with 900 water molecules per TA/lipid molecule (99.9 mol% water), were used to reflect TA/lipid seeds present in the initial (dilute) stages of self-assembly. Systems modelled with DMG-PEG_{400} (9 PEG monomers) were used to study the general effects of water and TA concentration, whereas the simulations with DMG-PEG_{2000} (45 PEG monomers) provided specific insights into a composition that is fully consistent with the 2:1 TA:DMG-PEG (10 mM) cubic assembly that has a final TA-to-DMG-PEG molar ratio of 2.7:1 (Fig. S3). This approach allowed for a systematic investigation of the fundamental interactions responsible for the formation of the solid-state-like, assembled materials. By varying the lipid-PEG chain length from 9 to 45 monomers, the influence of PEG chain length on the final structures was also assessed.

The Packmol program (version 20.14.2)[6,7] was used to prepare models for MD. Lipid and TA molecules were randomly distributed with intermolecular distances of at least 1 nm. Water molecules were added at distances greater than 0.2 nm from other molecular components. The coordinates for DMG-PEG_{400} and DMG-PEG_{2000}, used during packing, were generated by the CHARMM-GUI web server[8] in a π-helical conformation along the principal molecular axis. TA was constructed using the VMD 1.9.3 software[9] with an initial conformation having galloyl arms planar to the central glucose moiety (Fig. S7B). These structural features were artificially introduced to avoid steric clashes and excessively large periodic box sizes during model generation, and they dissipated rapidly into more energetically favorable conformations within the first few picoseconds of MD equilibration.

In total, 15 atomic models with explicit solvent were constructed and labeled based on TA:lipid molar ratios ranging from 0:1 to 3:1 (Fig. S7A and Table S3). To distinguish between systems with different hydration levels, subscripts *fh* (full hydration with 900 water molecules per TA/lipid) and *mh* (moderate hydration with 150 water molecules per TA/lipid) are used, whereas dehydrated models with 15 water molecules per TA/lipid have no subscript. The two systems simulated with DMG-PEG_{2000} that most closely represent the composition of 2:1 TA/DMG-PEG cubosomes have been labeled as 2.7:1 and $2.7{:}1^{mh}$.

MD: Force field parameters, equilibration protocol, and simulation settings

All-atom classical MD simulations were performed using the graphical processing unit (GPU) accelerated NAMD (version 2.14) program.[10] The CHARMM36 lipid force field,[11] CHARMM general force field (CGenFF) (version 4.0),[12] CHARMM carbohydrate force field,[13] and CHARMM-modified version of the TIP3P water model[14,15] were all employed. In addition, parameters adapted from the recently improved CHARMM lignin parameter set of Vermaas et al.[16] were used for galloyl groups and galloyl–carbohydrate interlinkages.

Models were equilibrated from disordered mixtures to prevent assemblies from becoming kinetically trapped due to packing bias. Without a priori knowledge of the assembled material

configurations, we employed a gentle equilibration protocol to ensure the final structures accurately represent equilibrium self-assembled structures. For the low and moderately hydrated systems, initial equilibration was performed in the canonical (*NVT*) ensemble using cubic unit cells roughly 2–3 times larger than the target sizes (Table S3). The periodic unit cell volume was gradually compressed using 50–160 individual consecutive 1 ns *NVT* simulations at 800 K, reducing the cell dimensions by 0.1 nm with each run until the atomic density became approximately uniform throughout. The high temperature allowed molecules sufficient kinetic energy to randomly mix during the unit cell compression process. Each simulation was minimized for 10,000 steps before running MD for 1,000,000 steps (1 ns). Once the target box size was reached (Table S1), atomic velocities were reset using a Boltzmann-weighted ensemble at 500 K and *NVT* equilibration continued for 1 ns. For all *NVT* simulations, velocities were reassigned to 800 or 500 K every 500 steps. Subsequently, the isothermal–isobaric (*NPT*) ensemble was used to equilibrate the pressure to 1 atm while slowly cooling the temperature from 500 to 300 K at a rate of 0.1 K every 0.2 ns. Similar equilibration protocols have been used to explore self-assembling ionic liquid crystals.[17] Following the initial shrinking and cooling equilibrations, *NPT* simulations were conducted under ambient conditions of 1 atm and 303.15 K for 160 ns to collect data for analysis. For the highly hydrated systems, constituent molecules were placed in a 14 × 14 × 14 nm^3 box and solvated with a water density of approximately 1 g cm^{-3}. Production simulations began directly to equilibrate the assemblies and collect data, equivalent to the final step for the low and moderately hydrated models, under ambient *NPT* conditions of 1 atm and 303.15 K for 160 ns.

All *NVT* equilibration was performed with an integration timestep of 1.0 fs, and all *NPT* simulations were performed with an integration timestep of 2.0 fs. Nonbonded interactions were managed with a cutoff distance of 1.2 nm, a switching distance of 1.0 nm, and a pair list distance of 1.6 nm, updated every 10 steps. Electrostatics were handled using the Particle Mesh Ewald (PME) method[18] with an interpolation order of 6 and a grid spacing of 0.1 nm. Rigid bonds for all hydrogen-involving bonds were enforced using the ShakeH algorithm of NAMD. Temperature was maintained using Langevin dynamics with a damping coefficient of 1.0 ps^{-1}. For *NPT* simulations, isotropic pressure coupling was controlled using the Nosé–Hoover Langevin piston method,[19,20] targeting a pressure of 1.01325 bar (1 atm) with a piston oscillation period of 50 fs and a piston decay time of 25 fs. Pressure was calculated using a hydrogen-group-based pseudo-molecular virial and kinetic energy approach, as this method results in less fluctuations and is required when using rigid ShakeH bonds.

The final 60 ns of the ambient *NPT* simulations were used for data analysis after discarding the first 100 ns for equilibration. Frames were saved to a trajectory every 10 ps. Analysis focused on the solid-state low hydration systems with varying TA concentrations (Fig. S2, S8–S13) to best reflect the experimentally characterized systems (Fig. S3). The VMD 1.9.3 software[9] was used for visualization, atomic rendering, calculating 1D (Fig. 2A) and 2D (Fig. S13) mass densities via the DensityCalculator TCL code,[21] and for creating volumetric occupancy maps to color water molecules in Movie S1. The MDAnalysis[22] and MDVWhole[23] python packages were used for trajectory post-processing and analysis.

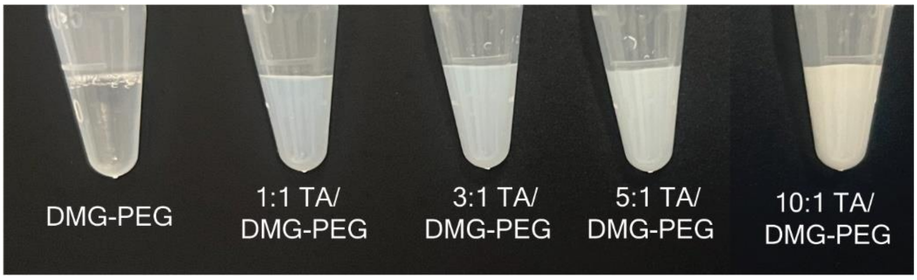

Figure S1. Photograph of the DMG-PEG NPs and TA/DMG-PEG cubosomes assembled from TA and DMG-PEG at different molar ratios. The concentration of DMG-PEG used for particle preparation was 2 mM.

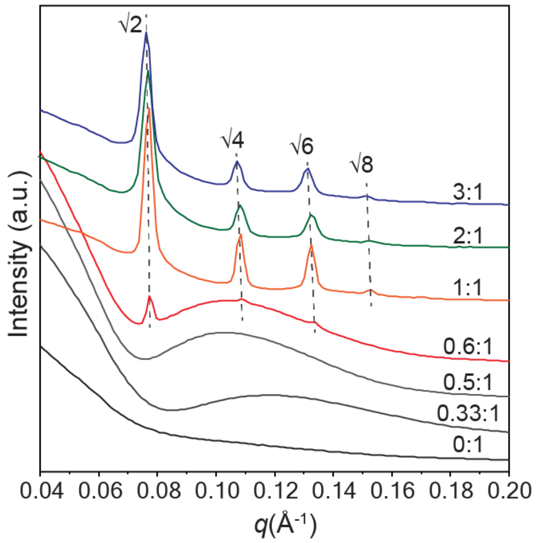

Figure S2. 1D diffraction SAXS patterns of TA/DMG-PEG cubosomes prepared from different molar ratios between TA and DMG-PEG (from 0:1 to 3:1). The concentration of DMG-PEG used for particle preparation was 10 mM.

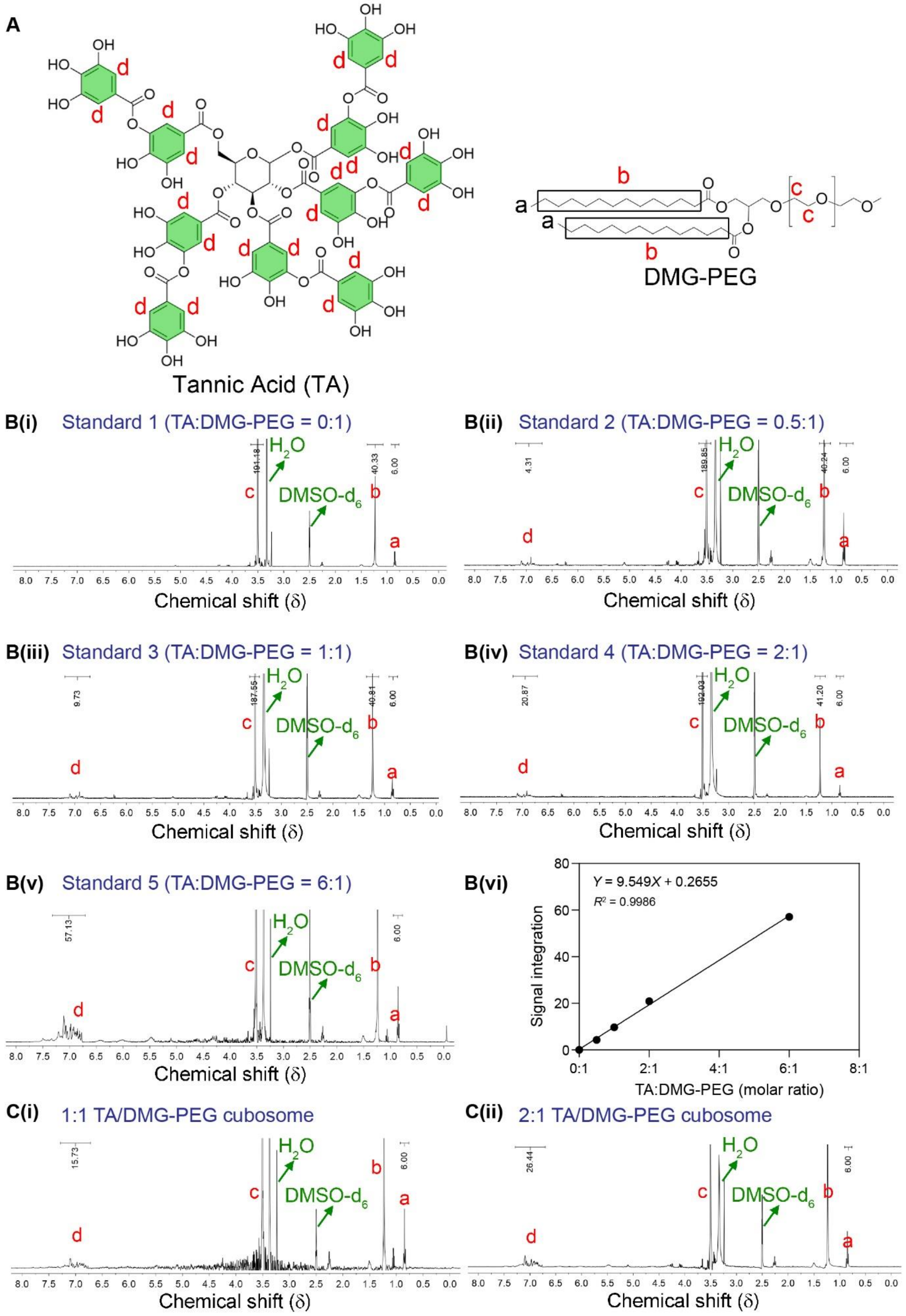

Figure S3. ^{1}H NMR measurements to determine the proportion of TA and DMG-PEG in the TA/DMG-PEG cubosomes. (**A**) Chemical structures of TA and DMG-PEG. (**B**) ^{1}H NMR spectra (**B**(i)–**B**(v)) and standard curve (**B**(vi) obtained from the signal integration of peak d) of

TA and DMG-PEG standards prepared by dissolving TA and DMG-PEG at specific molar ratios in DMSO-d6. (**C**) ^{1}H NMR spectra of TA/DMG-PEG cubosomes assembled from TA and DMG-PEG at different precursor molar ratios (1:1 TA:DMG-PEG (**C(i)**) and 2:1 TA:DMG-PEG (**C(ii)**)). The concentration of DMG-PEG used for particle preparation was 2 mM. Based on the standards, the final molar ratios of TA to DMG-PEG in **C(i)** and **C(ii)** were approximately 1.6:1 and 2.7:1.

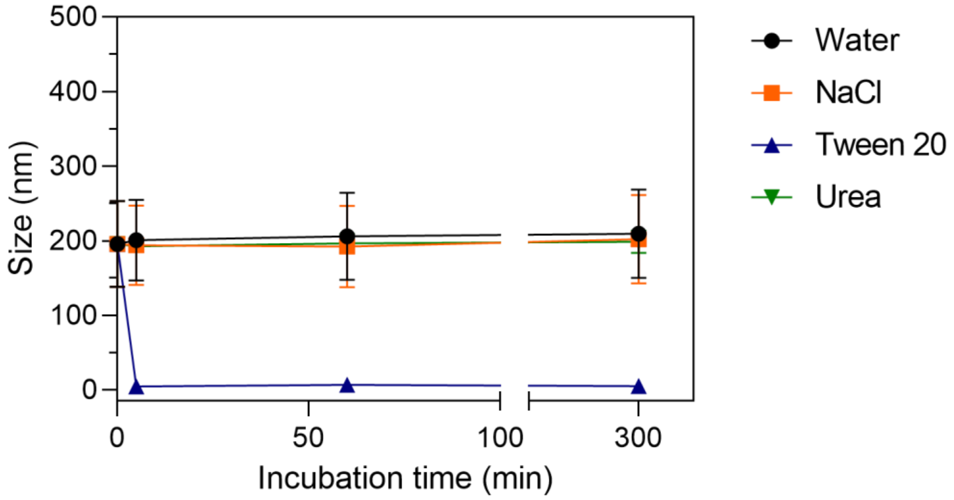

Figure S4. Stability (as measured by changes in particle size) of TA/DMG-PEG cubosomes (10:1 TA:DMG-PEG, 2 mM DMG-PEG) in different solutions over time. Data are shown as mean ± SD (n = 3 independent replicates).

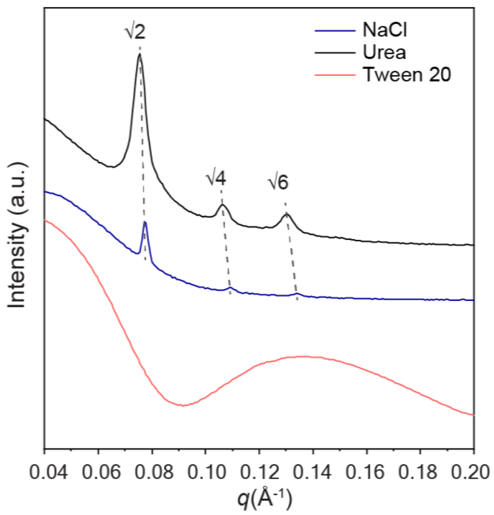

Figure S5. 1D diffraction SAXS patterns of TA/DMG-PEG cubosomes (1:1 TA:DMG-PEG, 10 mM DMG-PEG) after incubation with NaCl, urea, or Tween 20.

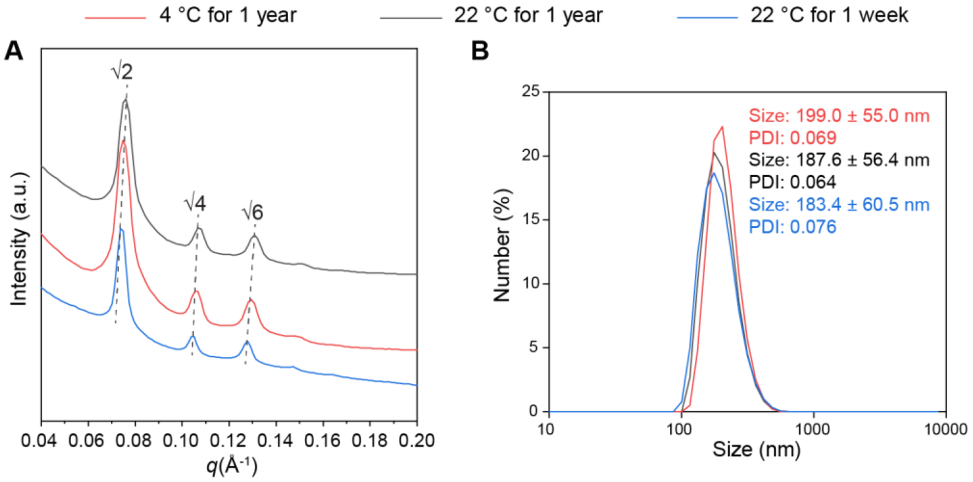

Figure S6. (**A, B**) 1D diffraction SAXS patterns (**A**) and size distribution (**B**) of TA/DMG-PEG cubosomes (10:1 TA:DMG-PEG, 2 mM DMG-PEG) after storage at 4 °C for 1 year or 22 °C for 1 week or 1 year. PDI, polydispersity index.

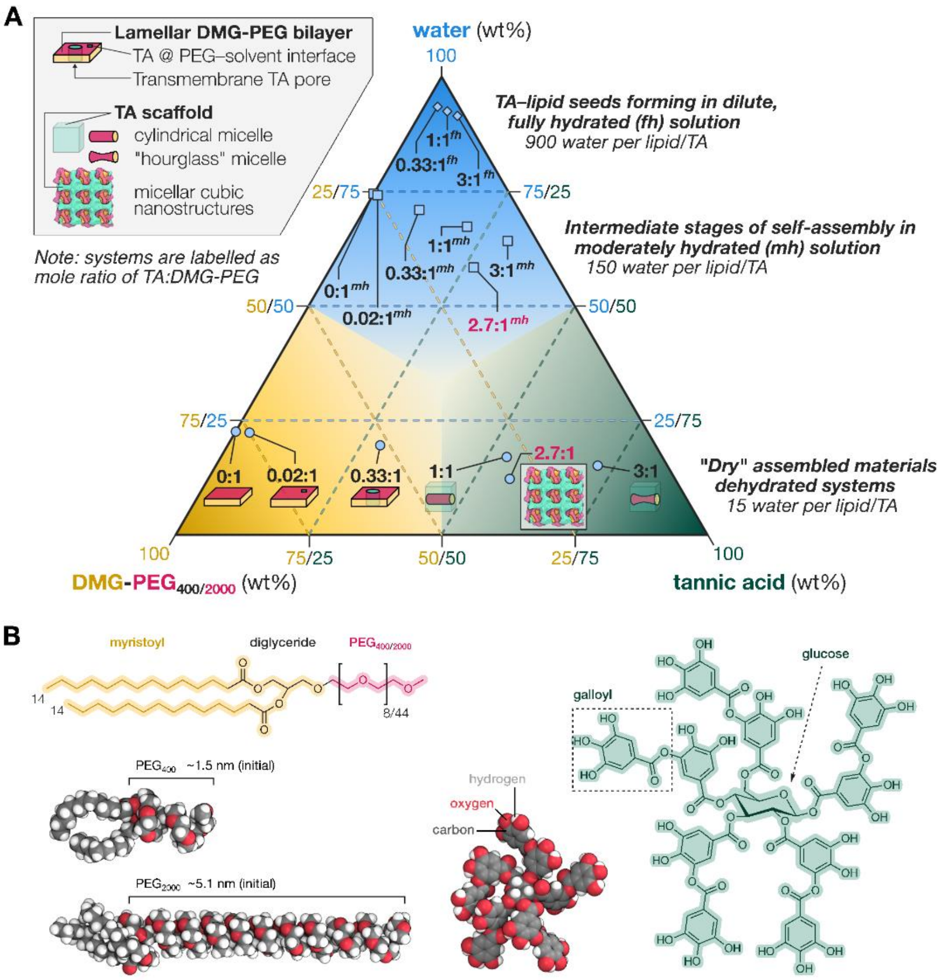

Figure S7. Summary of systems modeled by MD. (**A**) Phase diagram highlighting the range of compositions investigated by varying the relative concentrations of TA, DMG-PEG, and water. Data points are relative to the weight percentage (wt%) of each component in the ternary plot, whereas ratio labels indicate TA:DMG-PEG molar ratios. (**B**) Initial conformations and chemical structures of the constituent molecules simulated in the TA–lipid assemblies. The DMG-PEG$_{400}$ lipids (9 PEG monomers, 414 atoms) and DMG-PEG$_{2000}$ lipids (45 PEG monomers, 162 atoms) have a π-helical conformation along their principal molecular axis. The TA molecules (174 atoms) feature galloyl arms approximately planar with the central β-D-glucose moiety. TA is modeled with galloyl moieties linked via meta-depside bonds. Atomistic space-filling representations depict carbon in grey, oxygen in red, and hydrogen in white.

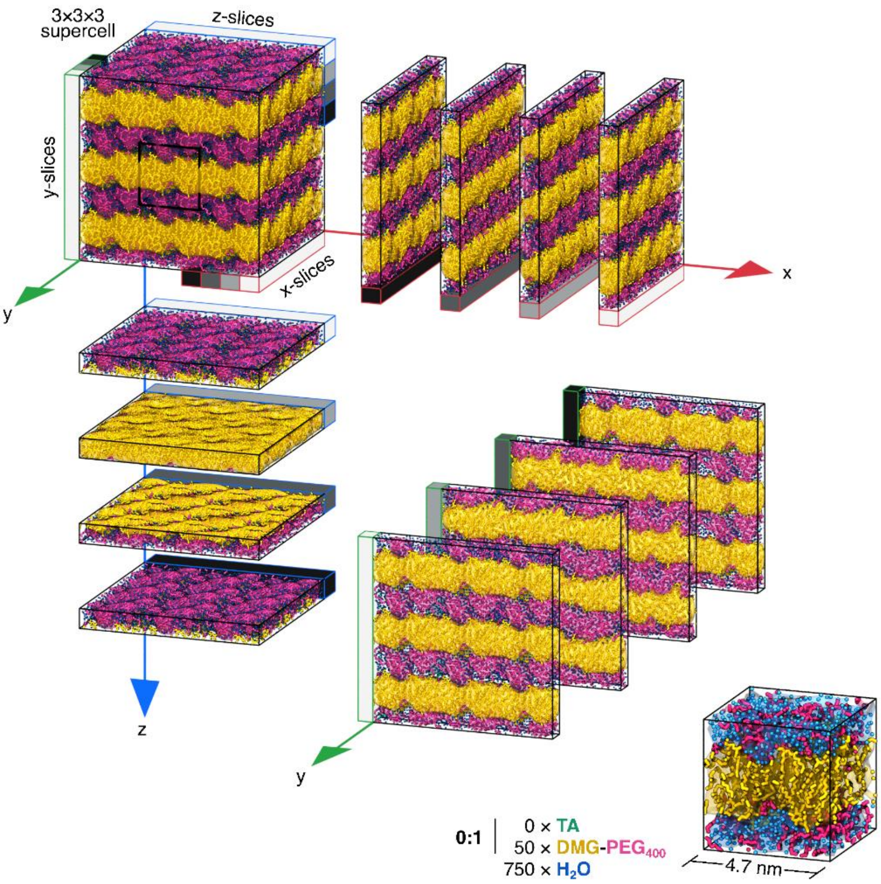

Figure S8. Structures from lipid-only MD (0:1) – 2D lamellar bilayers. Representative atomistic snapshots showcasing the final, equilibrated structure from MD simulations for the low hydration (15 water molecules per lipid) lipid-only system. The images illustrate the primitive unit cell (bottom right), a 3 × 3 × 3 supercell (top left), and 1.5 nm thick molecular slices along the Cartesian axes. The snapshots reveal that DMG-PEG$_{400}$ lipid molecules assemble into 2D lamellar bilayers with PEG regions exposed to the solvent. Lipid tails and diglyceride moieties are shown in yellow, PEG in pink, and water molecules in blue.

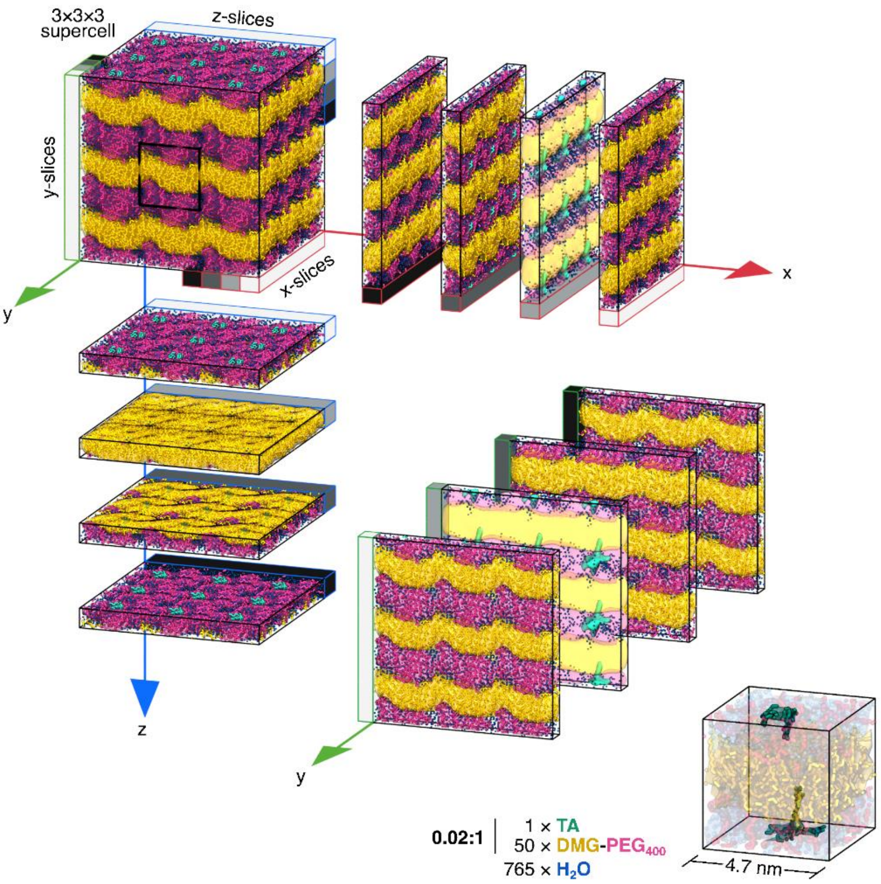

Figure S9. Structures from MD with low TA concentration (0.02:1) – TA at the PEG–solvent interface. Representative atomistic snapshots showcasing the final, equilibrated structure obtained from MD simulations for the low hydration system (15 water molecules per lipid/TA) containing lipid and a single TA molecule. The images illustrate the primitive unit cell (bottom right), a 3 × 3 × 3 supercell (top left), and 1.5 nm thick molecular slices along the Cartesian axes. The snapshots reveal that TA primarily integrates within the PEG–solvent interface of the 2D lamellar DMG-PEG$_{400}$ bilayers. Slices along the labeled *x*- and *y*-axes containing TA are shown using a coarse representation to better highlight the location of TA in the assembly. Lipid tails and diglyceride moieties are shown in yellow, PEG in pink, TA in cyan, and water molecules in blue.

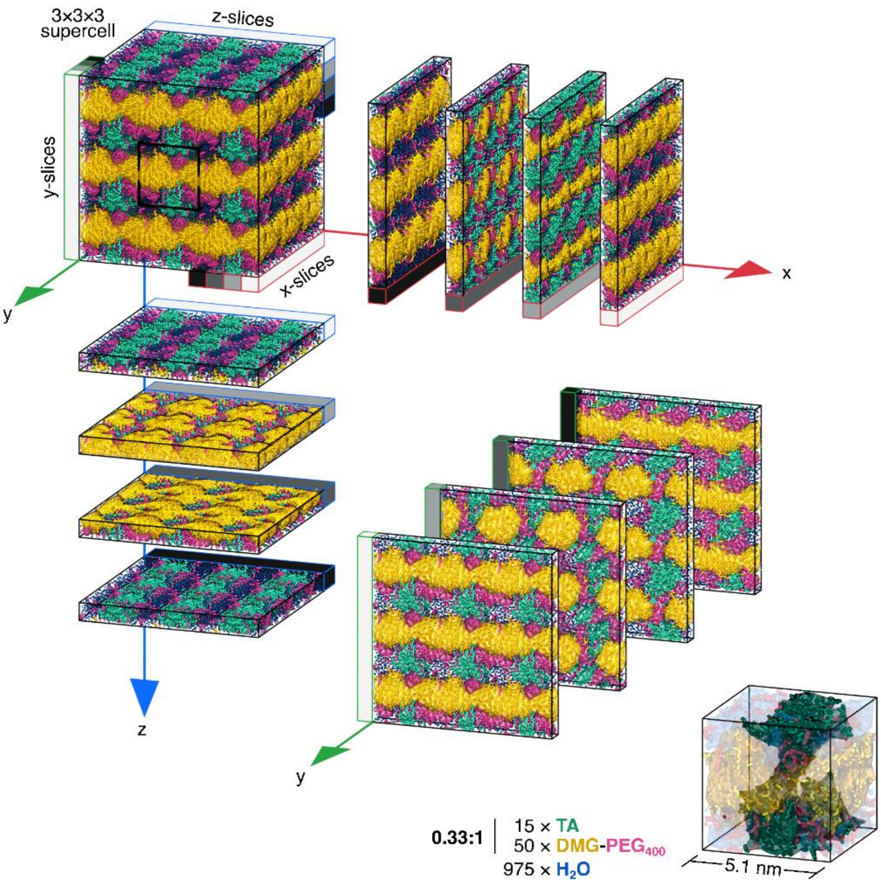

Figure S10. Structures from MD with moderate TA concentration (0.33:1) – Transmembrane TA pores. Representative atomistic snapshots showcasing the final, equilibrated structure obtained from MD simulations for the low hydration system (15 water molecules per lipid/TA) containing three times more lipid than TA. The images illustrate the primitive unit cell (bottom right), a 3 × 3 × 3 supercell (top left), and 1.5 nm thick molecular slices along the Cartesian axes. The snapshots reveal that as the concentration of TA increases, the 2D lamellar DMG-PEG_{400} bilayers exhibit a heterogeneous lateral density with transmembrane TA/DMG-PEG holes forming. Lipid tails and diglyceride moieties are shown in yellow, PEG in pink, TA in cyan, and water molecules in blue.

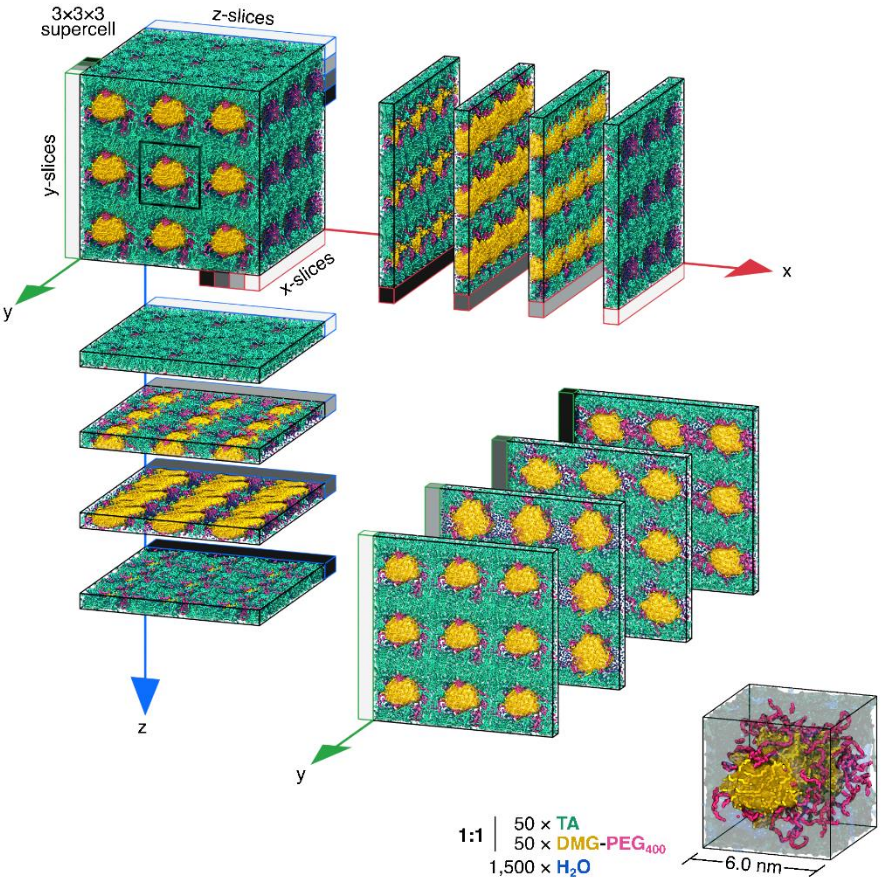

Figure S11. Structures from MD with equal TA:lipid ratio (1:1) – Cylindrical micelles. Representative atomistic snapshots showcasing the final, equilibrated structure obtained from MD simulations for the low hydration system (15 water molecules per lipid/TA) containing equal amounts of TA and lipid. The images illustrate the primitive unit cell (bottom right), a 3 × 3 × 3 supercell (top left), and 1.5 nm thick molecular slices along the Cartesian axes. The snapshots reveal that an equal molar concentration of lipid and TA results in DMG-PEG$_{400}$ molecules coalescing to form pseudo-1D cylindrical micelle-like structures encased in a TA scaffold. Lipid tails and diglyceride moieties are shown in yellow, PEG in pink, TA in cyan, and water molecules in blue.

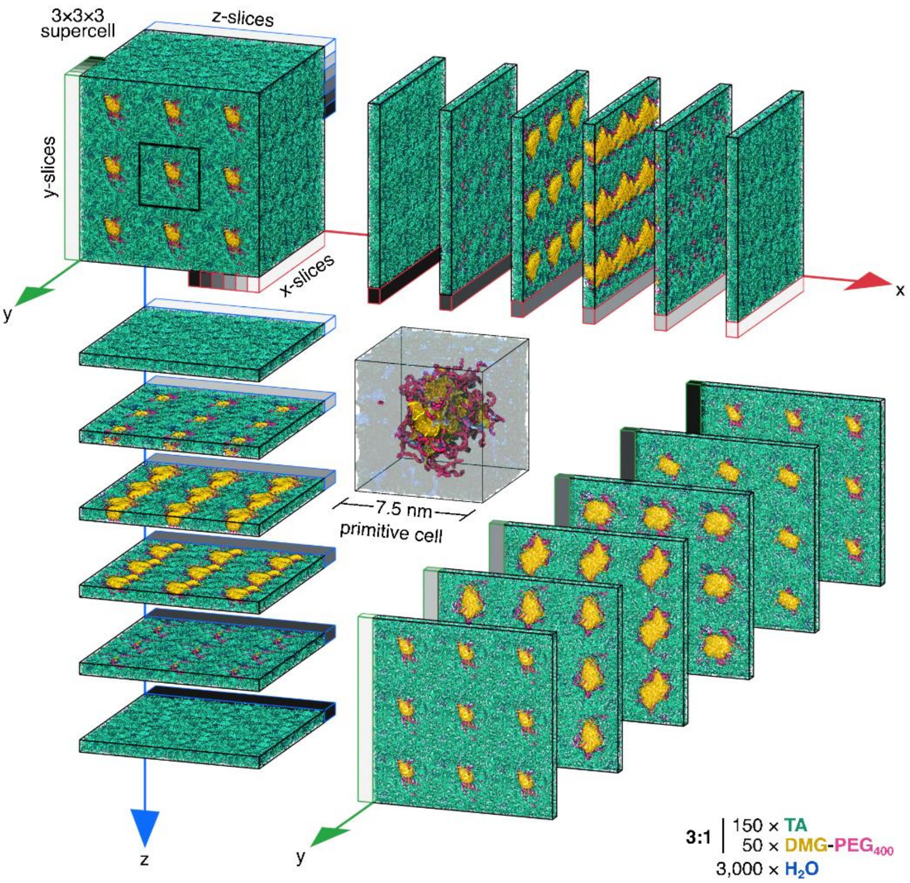

Figure S12. Structures from MD with high TA concentration (3:1) – "Hourglass" cylindrical micelles. Representative atomistic snapshots showcasing the final, equilibrated structure obtained from MD simulations for the low hydration system (15 water molecules per TA/lipid) containing three times more TA than lipid. The images illustrate the primitive unit cell (center), a 3 × 3 × 3 supercell (top left), and 1.5 nm thick molecular slices along the Cartesian axes. The snapshots reveal that at high molar concentrations of TA, the TA scaffold further segregates the DMG-PEG$_{400}$ molecules to form pseudo-1D micelle-like structures analogous to concave cylindrical (hourglass) structures, resulting in inhomogeneous lipid density along the *x*-, *y*-, and *z*-directions. Lipid tails and diglyceride moieties are shown in yellow, PEG in pink, TA in cyan, and water molecules in blue.

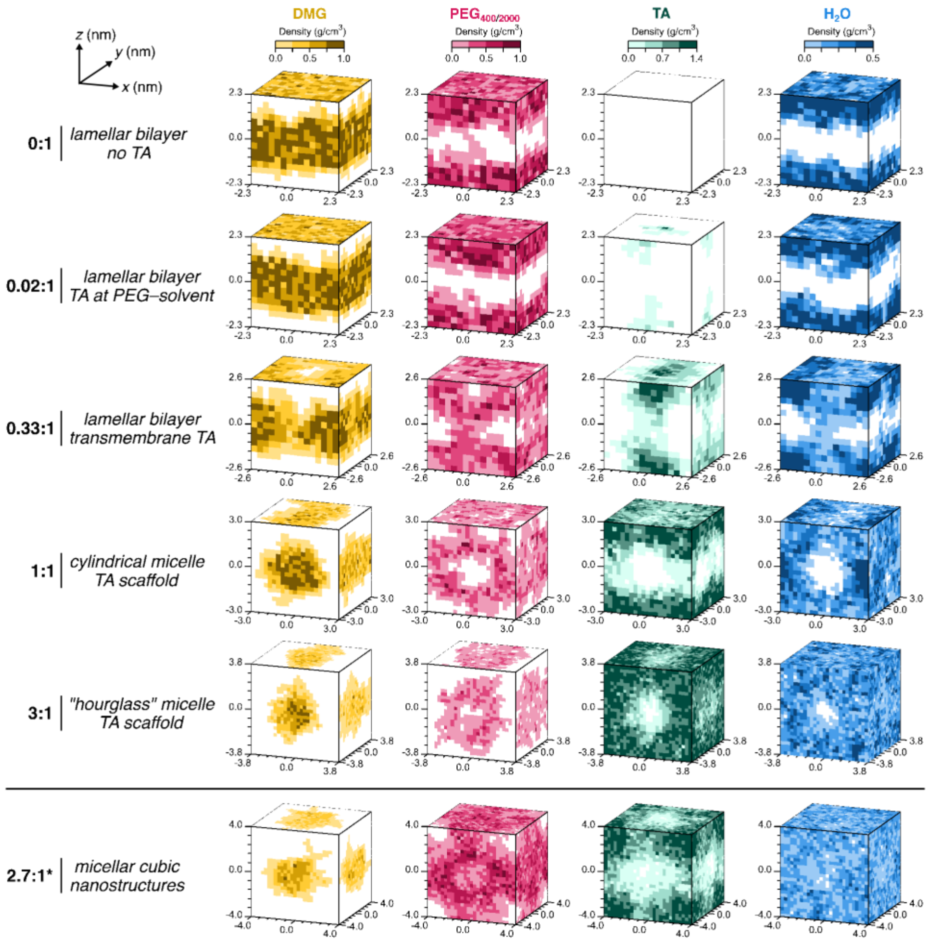

Figure S13. MD-generated 2D mass density plots for all low hydration systems. Densities are shown for each component: DMG lipid (yellow), PEG chains (pink), TA molecules (cyan), and water molecules (blue). The plots, projected onto cubic unit cell faces, display the *xy*, *xz*, and *yz* mass densities and provide a comprehensive view of the spatial distributions and interactions within the MD simulations. TA:lipid systems 0:1 and 0.02:1 exhibit planar lamellar bilayers with uniform lipid densities and highly solvated PEG_{400}/TA. The 0.33:1 system shows a hole in the DMG lipid *xy* density and thin *xz* regions that correlate with increased TA, PEG_{400}, and water densities, indicating a transmembrane pore. The 1:1 system forms a uniform DMG-PEG_{400} micellar cylinder encapsulated by TA and water. The 3:1 system shows that high TA concentrations lead to an inhomogeneous lipid distribution, mostly in the *y*-direction (as seen in the *xy* and *yz* densities), forming "hourglass" shaped cylindrical micelles across periodic boundaries. The 2.7:1* system features DMG-PEG_{2000} forming pancake-like micelles, an interconnected PEG network, an external TA scaffold, and continuous water density in all dimensions.

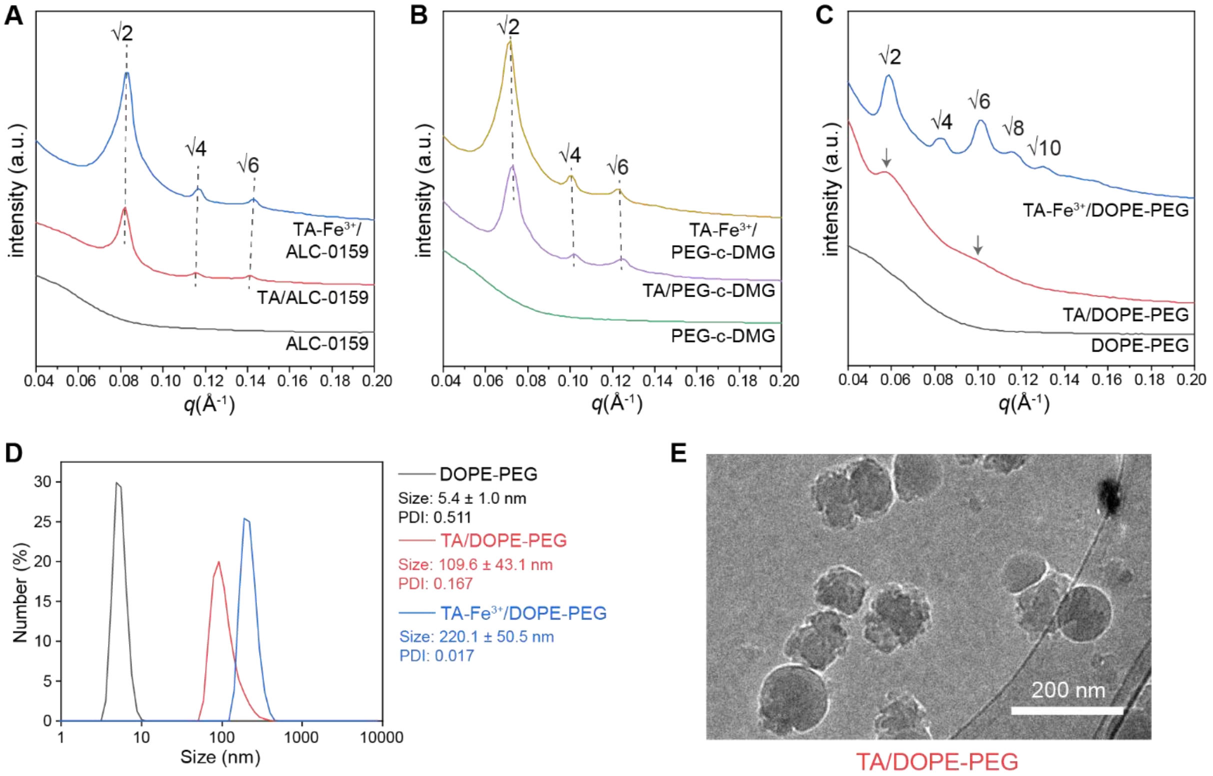

Figure S14. (**A–C**) 1D diffraction SAXS patterns of TA- and TA-Fe^{3+}-based cubosomes prepared using ALC-1059 (**A**), PEG-c-DMG (**B**), and DOPE-PEG (**C**). (**D**) Size distribution of DOPE-PEG, TA/DOPE-PEG, and TA-Fe^{3+}/DOPE-PEG NPs. (**E**) Cryo-electron microscopy image of TA/DOPE-PEG NPs. The broad bumps (indicated by the grey arrows) in the SAXS pattern of TA/DOPE-PEG NPs (**C**), their smaller size compared with TA-Fe^{3+}/DOPE-PEG cubosomes (**D**), as well as the loosely packed micelles inside TA/DOPE-PEG NPs without long-range order (**E**) collectively suggest that TA alone could not sufficiently modulate the mesophase of DOPE-PEG micelles into nanostructured NPs.

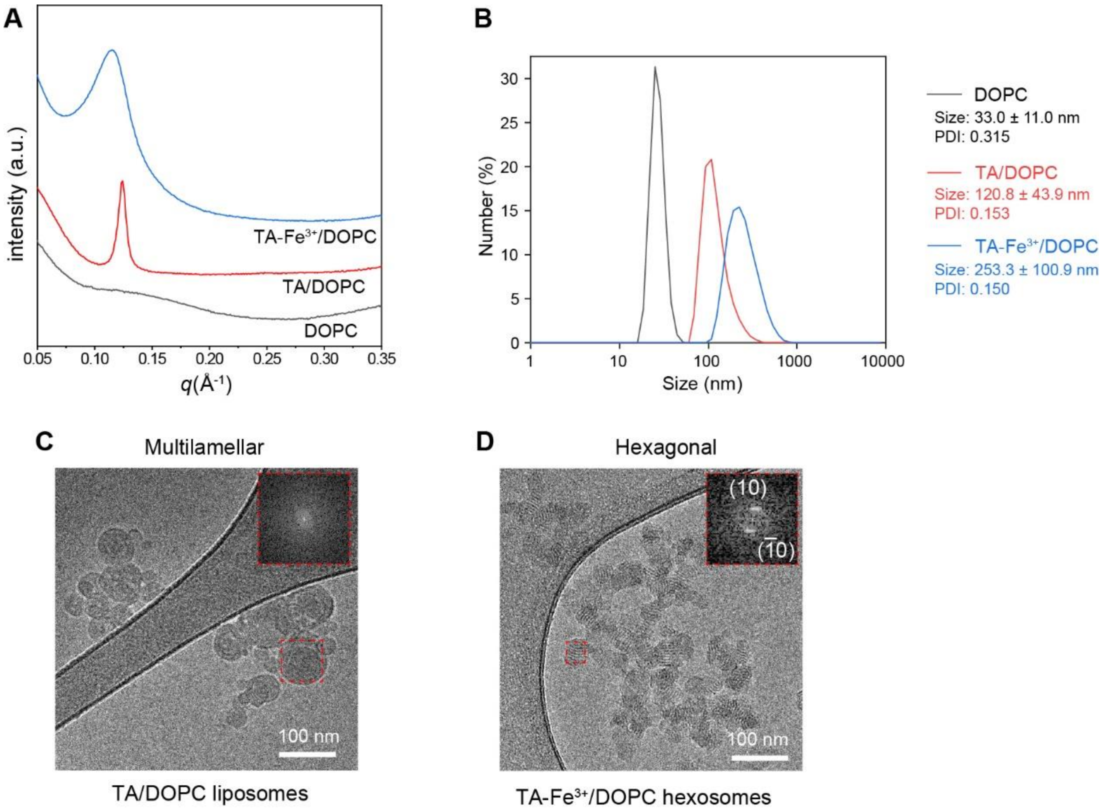

Figure S15. (**A**) 1D diffraction SAXS patterns of DOPC, TA/DOPC, and TA-Fe^{3+}/DOPC NPs. (**B**) Size distributions of DOPC, TA/DOPC, and TA-Fe^{3+}/DOPC NPs. (**C**, **D**) Cryo-electron microscopy images of TA/DOPC NPs with multilamellar phases (**C**) and TA-Fe^{3+}/DOPC NPs with hexagonal phases (**D**). Insets in (**C** and **D**) are fast Fourier transform images with assigned Miller indices for the section marked with a red frame. The concentric lamellae in TA/DOPC NPs suggest multilamellar nanostructures in the NPs. The oblate ellipsoid morphology of the TA-Fe^{3+}/DOPC NPs, the continuous lattice fringes inside the NPs, and the fast Fourier transform images of the lattice suggest hexagonal nanostructures in the TA-Fe^{3+}/DOPC NPs.[24]

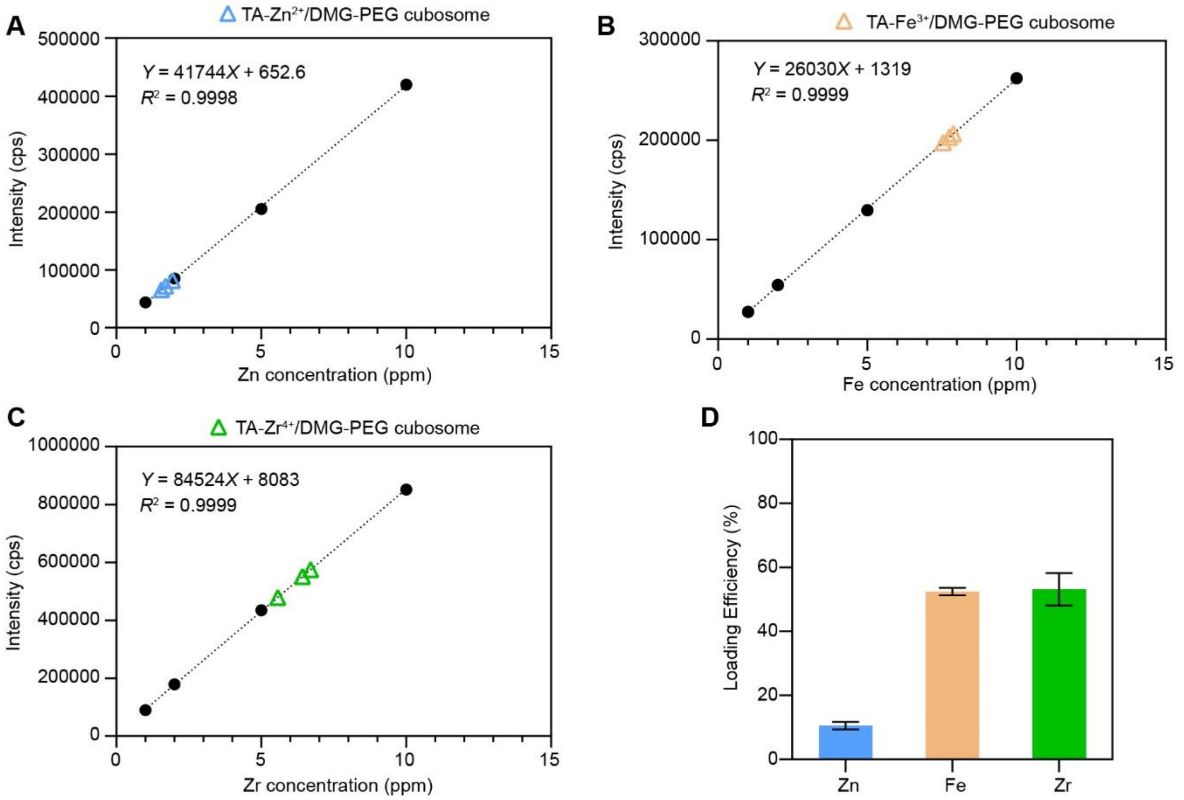

Figure S16. Quantification of metal loading in TA-metal ion/DMG-PEG cubosomes. (**A–C**) Calibration curves for Zn (213.857) (**A**), Fe (238.204) (**B**), and Zr (343.823) (**C**) measured by ICP-OES. Colored triangles represent measured intensities from TA-Zn^{2+}/DMG-PEG (1:1 TA:Zn^{2+}) (**A**), TA-Fe^{3+}/DMG-PEG (1:1 TA:Fe^{3+}) (**B**), and TA-Zr^{4+}/DMG-PEG (2:1 TA:Zr^{4+}) (**C**) cubosome samples, plotted against standard calibration curves. (**D**) Metal loading efficiency of TA-metal ion/DMG-PEG cubosomes calculated from ICP data. Data are shown as mean ± SD (n = 3 independent replicates).

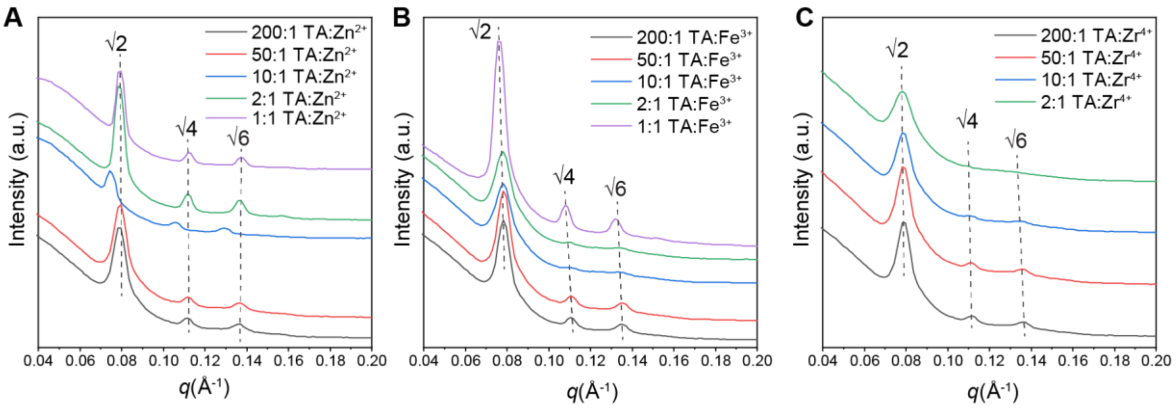

Figure S17. (**A–C**) 1D diffraction SAXS patterns of DMG-PEG-based MPN cubosomes prepared from TA and metal ions Zn^{2+} (**A**), Fe^{3+} (**B**), or Zr^{4+} (**C**) of different TA/metal ions molar ratios.

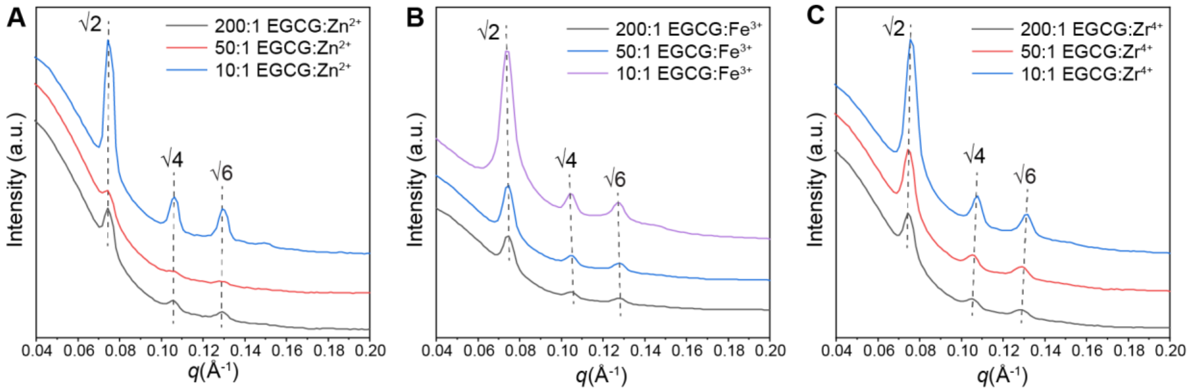

Figure S18. (**A–C**) 1D diffraction SAXS patterns of DMG-PEG-based MPN cubosomes prepared from EGCG and metal ions Zn^{2+} (**A**), Fe^{3+} (**B**), or Zr^{4+} (**C**) of different EGCG/metal ions molar ratios.

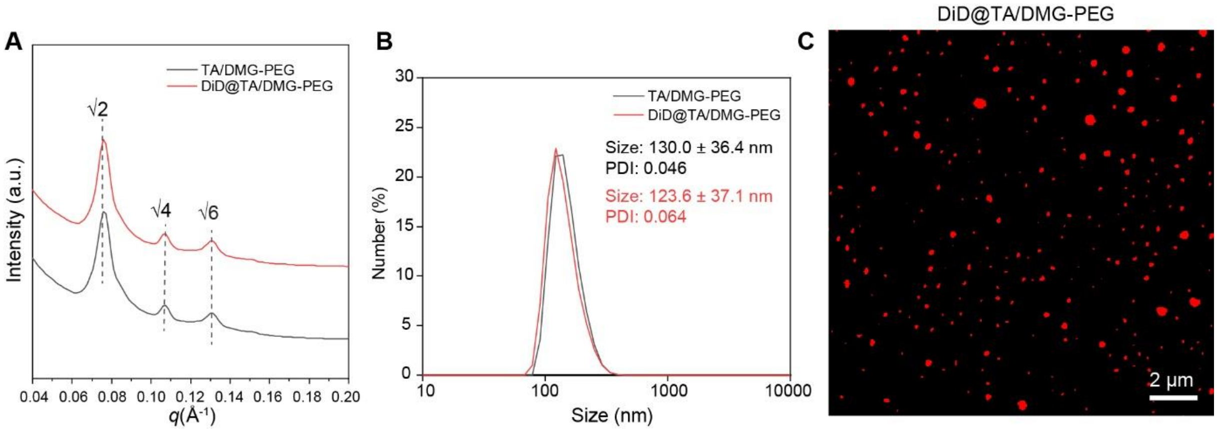

Figure S19. Characterization of DiD-loaded TA/DMG-PEG (DiD@TA/DMG-PEG) cubosomes. (**A, B**) 1D diffraction SAXS patterns (**A**) and size distributions (**B**) of TA/DMG-PEG and DiD@TA/DMG-PEG cubosomes. (**C**) Super-resolution (via lattice-SIM) microscopy images of DiD@TA/DMG-PEG cubosomes.

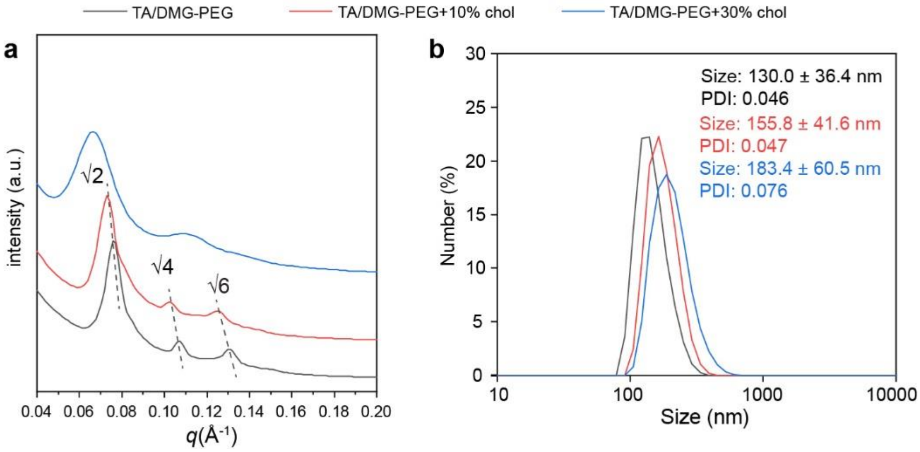

Figure S20. (**A, B**) 1D diffraction SAXS patterns (**A**) and size distributions (**B**) of TA/DMG-PEG+10% chol and TA/DMG-PEG+30% chol cubosomes prepared by respectively adding 10% and 30% cholesterol (chol) in the ethanol phase.

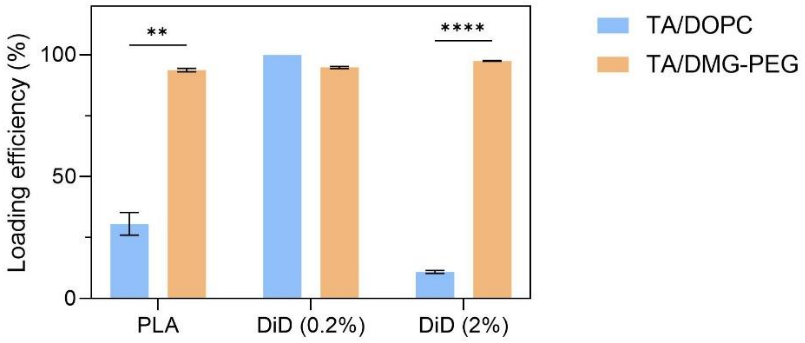

Figure S21. Comparison of cargo loading efficiency of TA/DOPC liposomes and TA/DMG-PEG cubosomes. The encapsulation efficiencies of PLA and DiD (0.2% and 2%) were quantified. Data are presented as mean ± SD (n = 3 independent replicates); $^{**}p < 0.01$, $^{****}p < 0.0001$, by unpaired two-tailed t-test.

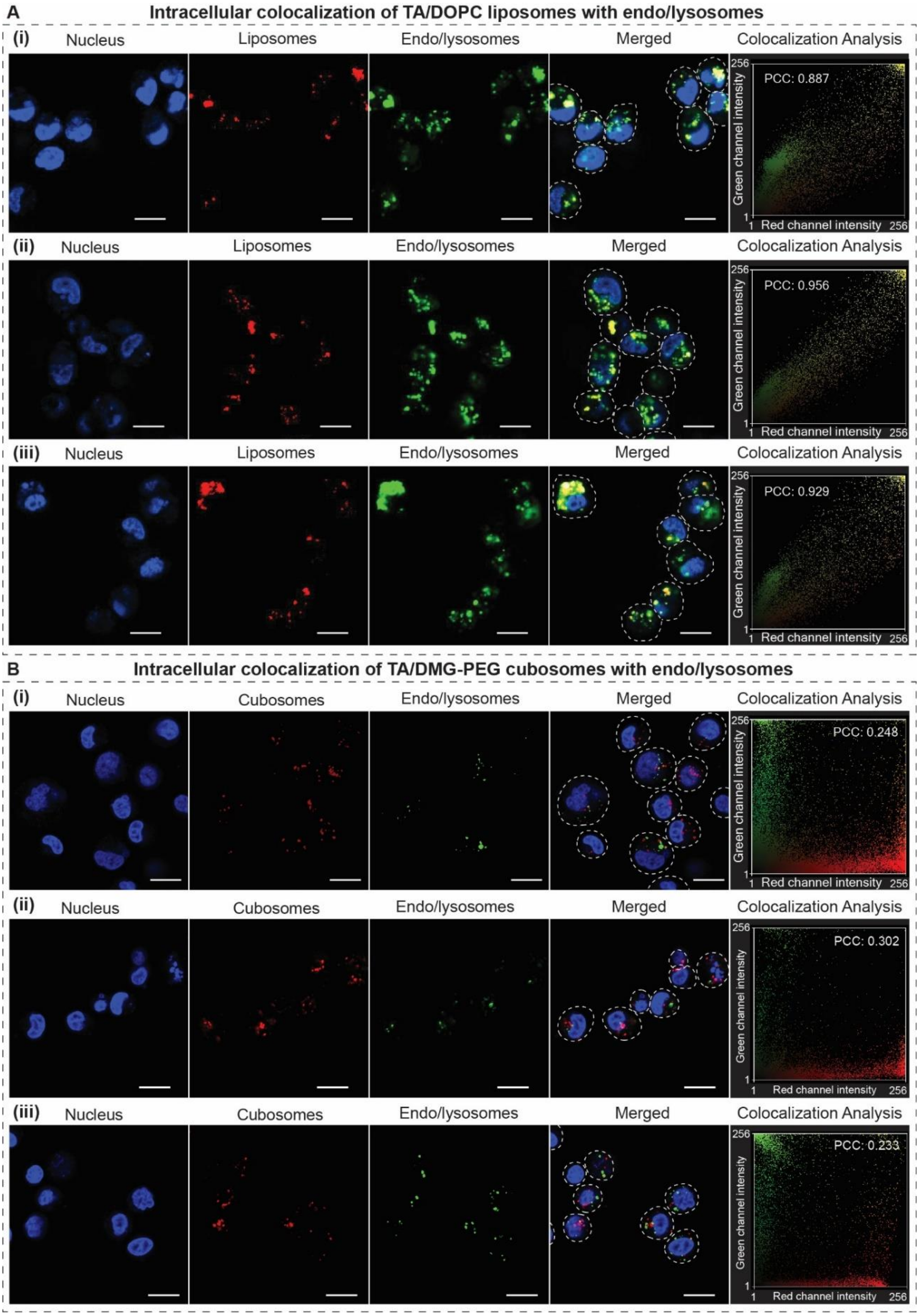

Figure S22. Intracellular colocalization of (**A**) TA/DOPC liposomes and (**B**) TA/DMG-PEG cubosomes with endo/lysosomes in MDA-MB-231 cells. Confocal microscopy images showing the intracellular localization of TA-based NPs with endo/lysosomes 4 h post incubation. NPs were labeled with DiD (red). Endo/lysosomes (green) were stained with LysoTracker Green DND-26. Nuclei were stained with Hoechst 33342 (blue). Scale bars are 15 µm. Intracellular colocalizations between NPs and endo/lysosomes were further validated by PCC analysis of endo/lysosomes (green channel) vs nanoparticles (red channel).

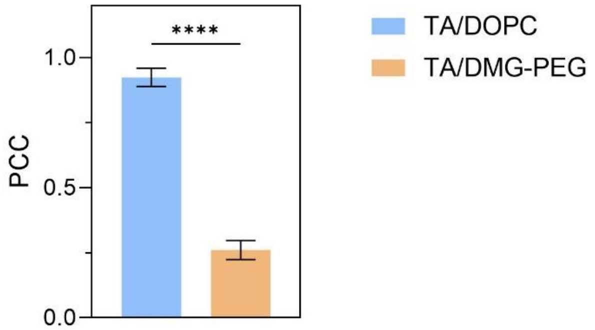

Figure S23. Quantification of colocalization between TA-based NPs and endo/lysosomes. TA/DOPC liposomes exhibited significantly higher colocalization (PCC ≈ 0.92) compared to TA/DMG-PEG cubosomes (PCC ≈ 0.26), suggesting enhanced endo/lysosomal escape of the TA/DMG-PEG cubosomes. Data are presented as mean ± SD (n = 3 independent replicates); ****$p < 0.0001$, by unpaired two-tailed t-test.

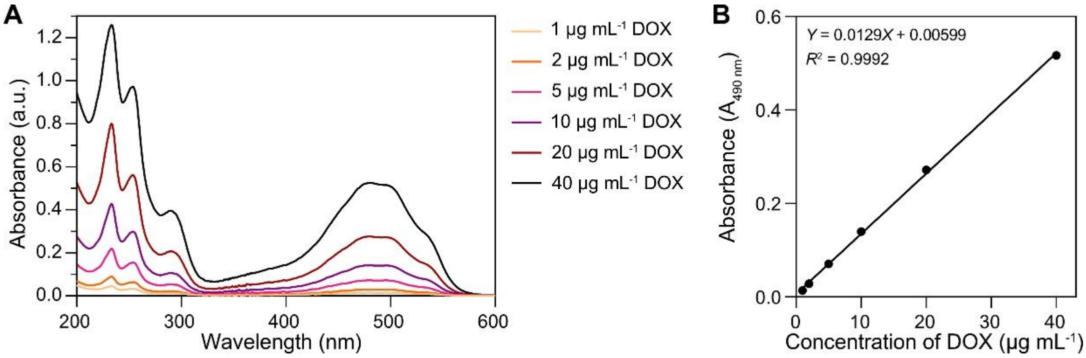

Figure S24. (**A**) UV–vis spectra of DOX aqueous solutions at varying concentrations. (**B**) Calibration curve of absorbance at 490 nm versus DOX concentration.

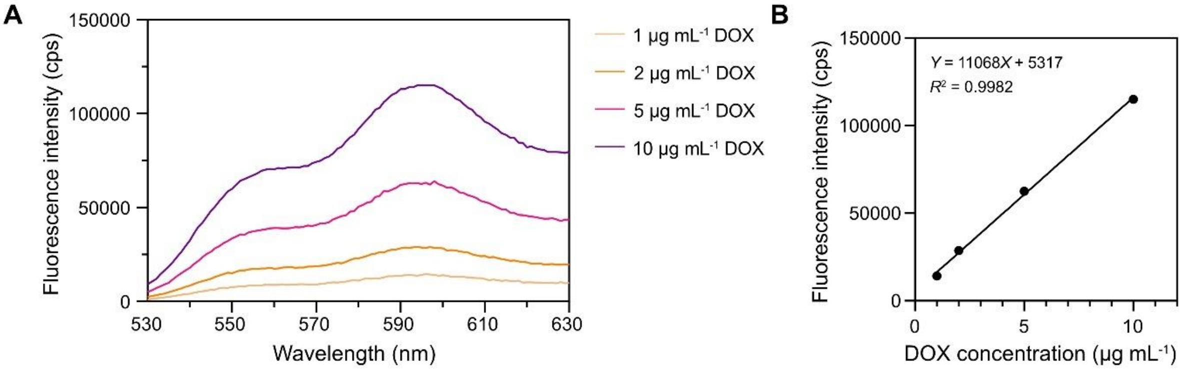

Figure S25. Fluorescence standard curve for DOX. (**A**) Emission spectra of DOX solutions at defined concentrations (λ_{ex} = 480 nm), showing characteristic fluorescence between 550–620 nm. (**B**) Calibration curve of emission fluorescence intensity at 595 nm versus DOX concentration.

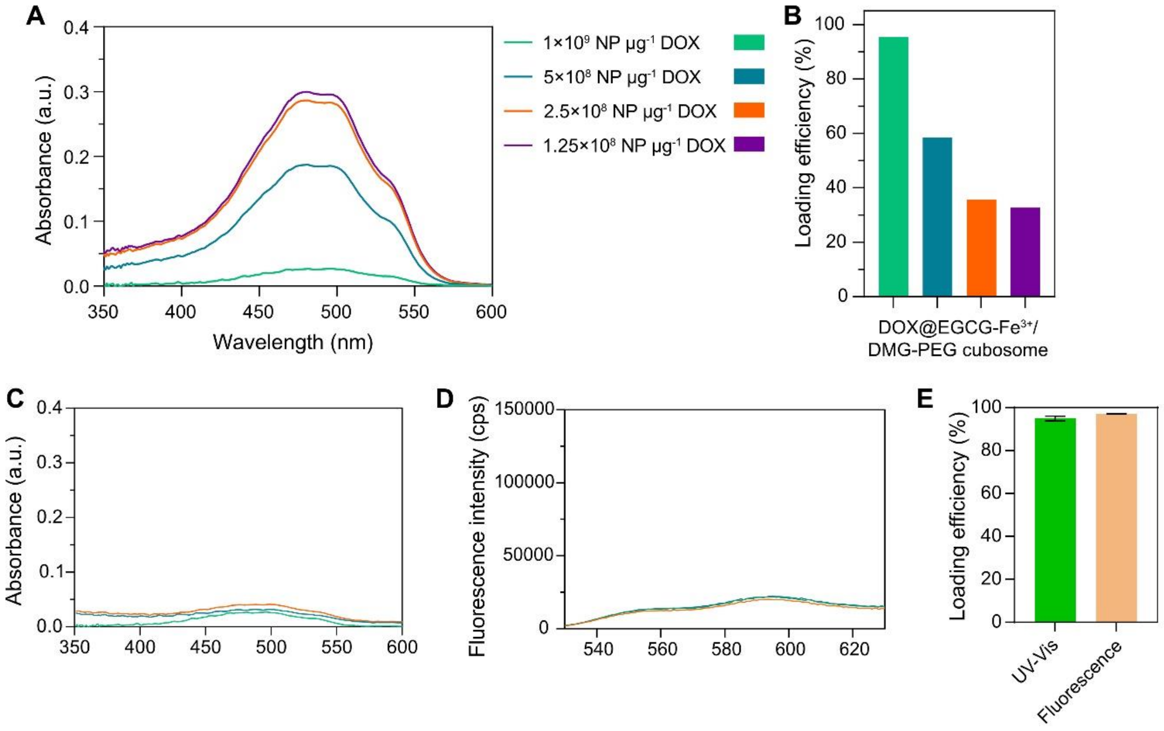

Figure S26. (**A**) UV–vis spectra of sample supernatants obtained by centrifuging DOX@EGCG-Fe^{3+}/DMG-PEG cubosomes after incubating EGCG-Fe^{3+}/DMG-PEG cubosomes with DOX at different cubosome-to-DOX ratios (NP per μg DOX). (**B**) DOX loading efficiency of DOX@EGCG-Fe^{3+}/DMG-PEG cubosomes. (**C**) UV–Vis spectra of the supernatants of DOX-loaded cubosome samples (1×10^9 NP per μg DOX) in 3 replicates. (**D**) Corresponding fluorescence emission spectra (λ_{ex} = 480 nm) of the samples. (**E**) Comparison of DOX loading efficiency of DOX@EGCG-Fe^{3+}/DMG-PEG cubosomes (1×10^9 NP per μg DOX) determined by UV–Vis and fluorescence spectroscopy, confirming good agreement between the two quantification methods. Data are shown as mean ± SD (n = 3 independent replicates).

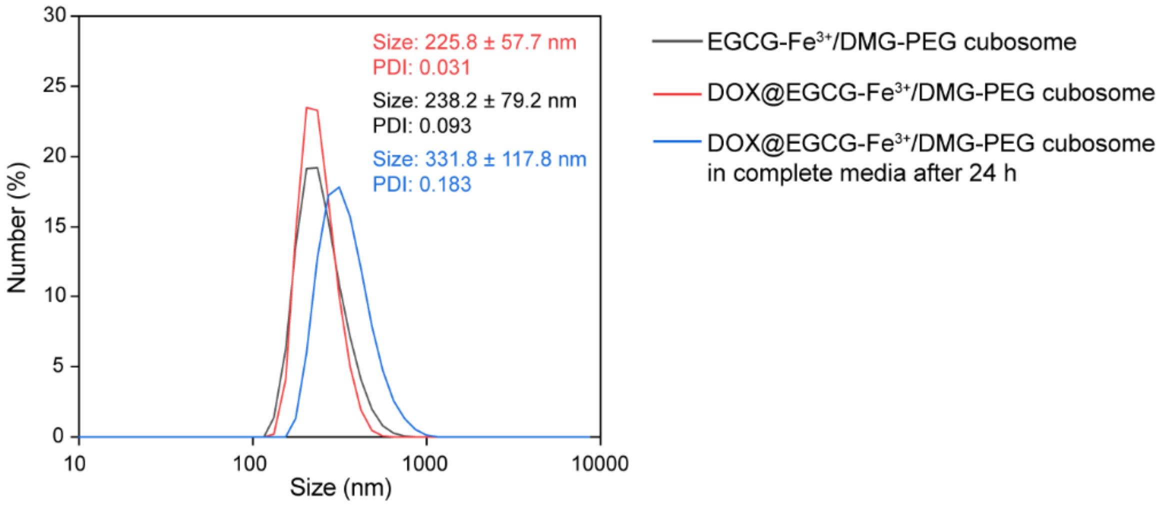

Figure S27. Size distributions of EGCG-Fe^{3+}/DMG-PEG cubosomes before and after loading with DOX, and DOX-loaded cubosomes after incubation in complete media (DMEM supplied with 10% FBS) for 24 h.

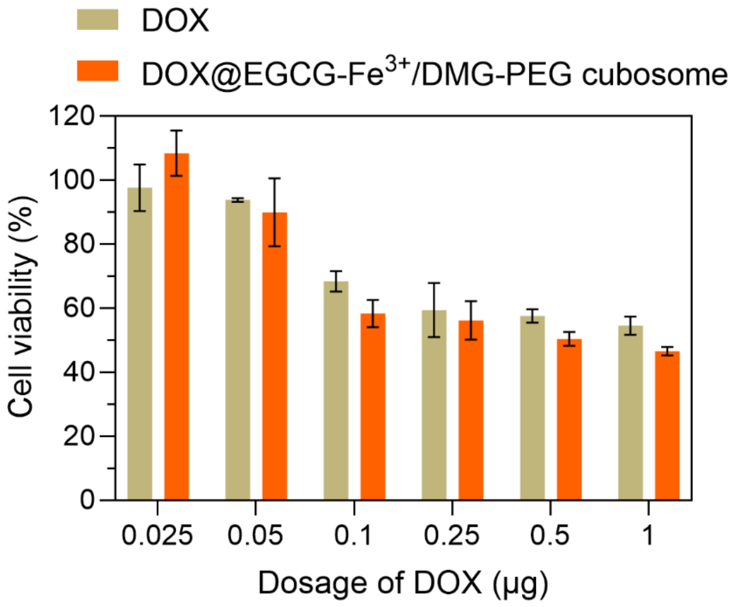

Figure S28. Viability of MDA-MB-231 cells after incubation with free DOX or DOX@EGCG-Fe^{3+}/DMG-PEG cubosomes at different drug dosages. Cell viability (%) was normalized to the cell only control group. Data are shown as mean ± SD (*n* = 3 independent replicates).

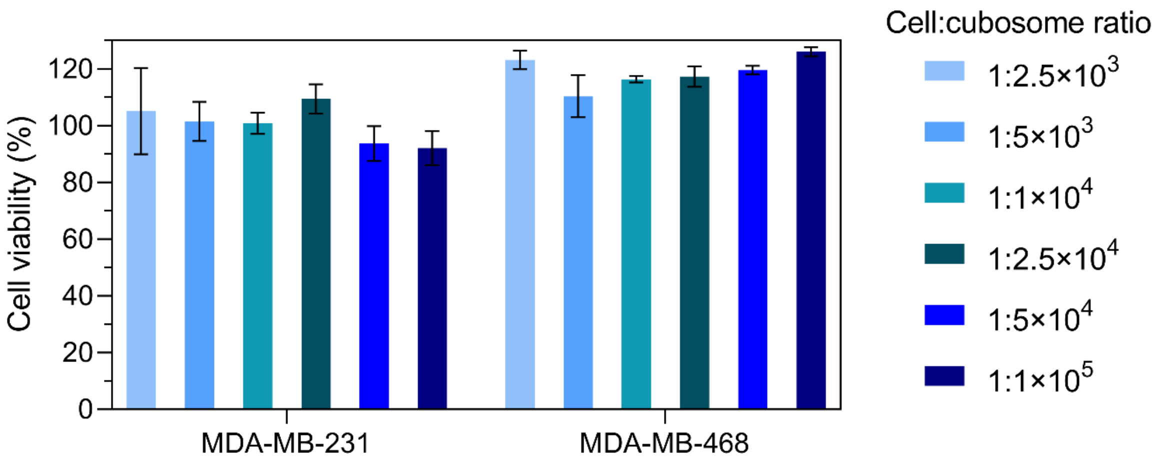

Figure S29. Viability of MDA-MB-231 and MDA-MB-468 cells after treatment of EGCG-Fe^{3+}/DMG-PEG cubosomes at different cell-to-cubosome ratios. Cell viability (%) was normalized to the cell only control group. Data are shown as mean ± SD (n = 3 independent replicates).

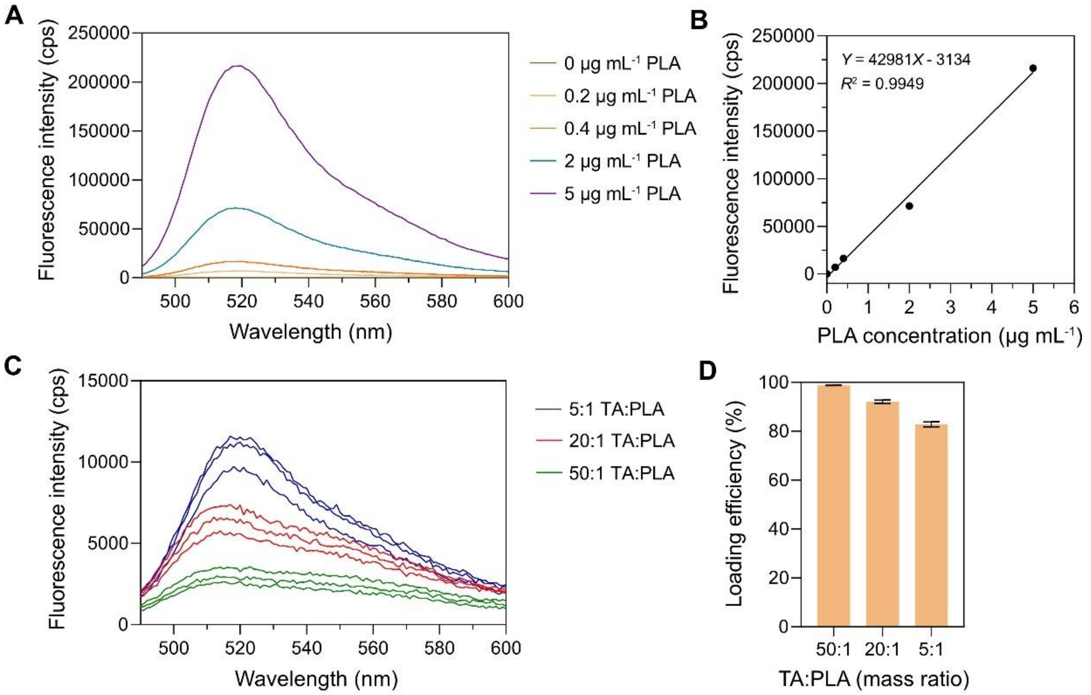

Figure S30. Fluorescence-based quantification of FITC-PLA loading in TA-based cubosomes. (**A**) Fluorescence emission spectra of FITC-PLA solutions at various concentrations (λ_{ex} = 480 nm). (**B**) Standard curve of fluorescence intensity versus FITC-PLA concentration. (**C**) Fluorescence emission spectra of supernatants collected from cubosome formulations at varying TA:PLA mass ratios (50:1, 20:1, and 5:1). PLA@TA/DMG-PEG cubosome samples at TA:PLA mass ratios of 50:1, 20:1, and 5:1 were diluted 2×, 20×, and 100×, respectively, before measurement. (**D**) Loading efficiencies of FITC-PLA based on the fluorescence measurements. Data are shown as mean ± SD (n = 3 independent replicates).

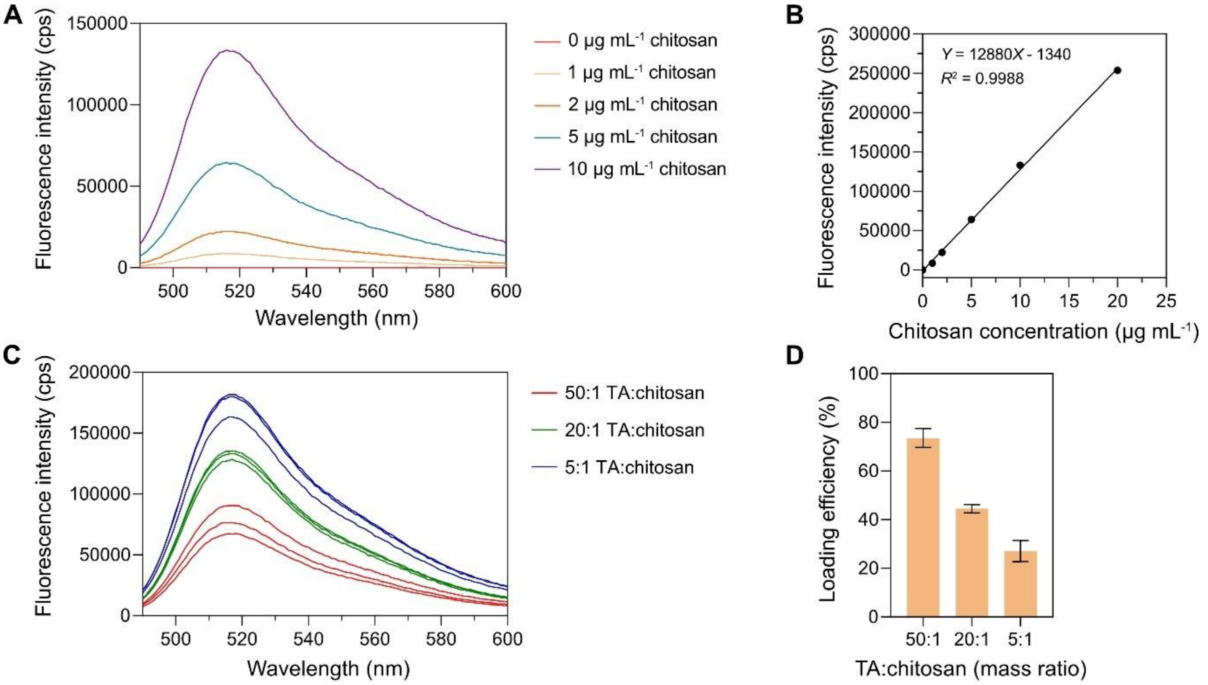

Figure S31. Fluorescence-based quantification of FITC-chitosan loading in TA-based cubosomes. (**A**) Fluorescence emission spectra of FITC-chitosan solutions (dissolved in citrate buffer (100 mM, pH 6)) at various concentrations (λ_{ex} = 480 nm). (**B**) Standard curve of fluorescence intensity versus FITC-chitosan concentration. (**C**) Fluorescence emission spectra of supernatants collected from cubosome formulations at varying TA:chitosan mass ratios (50:1, 20:1, and 5:1). Chitosan@TA/DMG-PEG cubosome samples at mass ratios of 20:1 and 5:1 were diluted 3×, and 10×, respectively, before measurement. (**D**) Loading efficiencies of FITC-chitosan based on the fluorescence measurements. Data are shown as mean ± SD (*n* = 3 independent replicates).

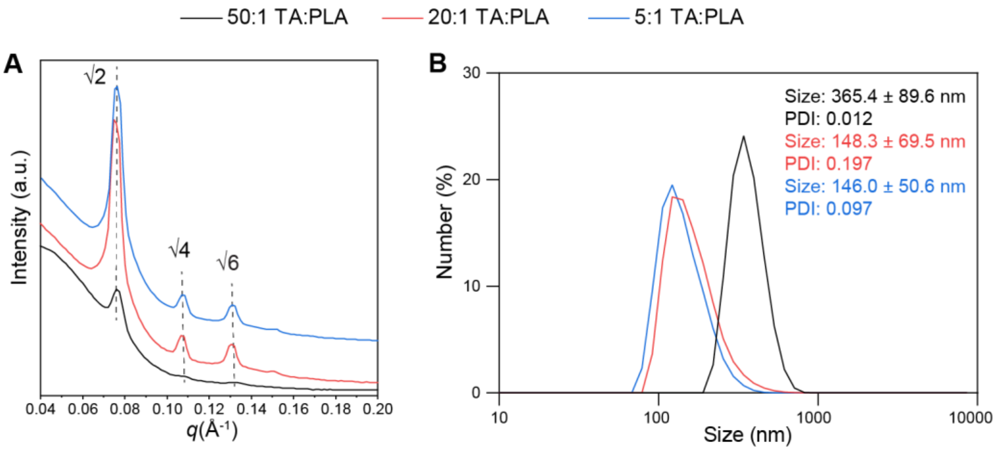

Figure S32. (**A, B**) 1D diffraction SAXS patterns (**A**) and size distribution (**B**) of PLA@TA/DMG-PEG cubosomes prepared using TA and PLA at different mass ratios.

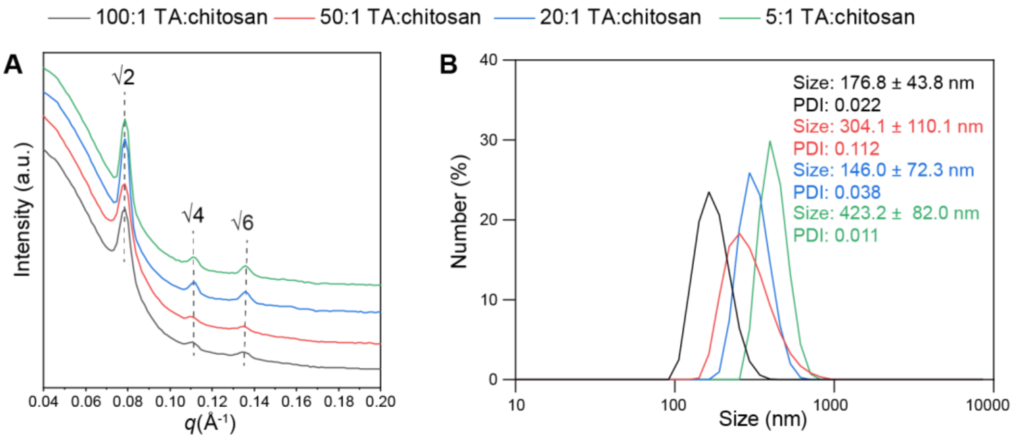

Figure S33. (**A, B**) 1D diffraction SAXS patterns (**A**) and size distribution (**B**) of chitosan@TA/DMG-PEG cubosomes prepared using TA and chitosan at different mass ratios.

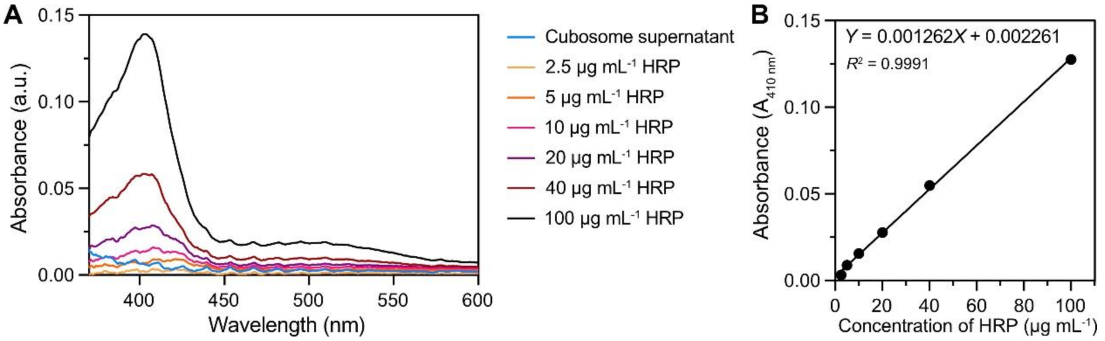

Figure S34. (**A**) UV–vis spectra of HRP aqueous solution at varying concentrations and sample supernatant obtained by centrifuging HRP@TA/DMG-PEG cubosomes. (**B**) Calibration curve of absorbance at 410 nm versus HRP concentration. Based on the standard curve, the concentration of HRP in HRP@TA/DMG-PEG cubosomes was 16.1 μg mL^{-1}.

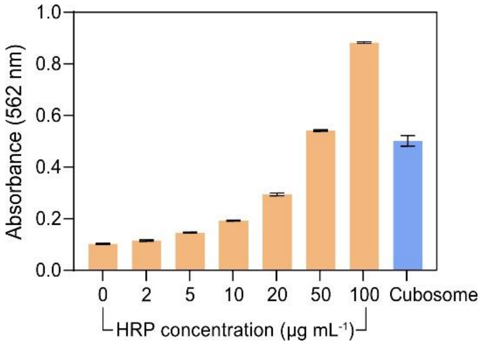

Figure S35. Standard curve showing the absorbance at 562 nm for HRP solutions at varying concentrations (orange bars), alongside a purified TA/DMG-PEG cubosome sample (5×10^7 μL^{-1}) without HRP loading (blue bar). Data are shown as mean ± SD (n = 3 independent replicates). Despite conducting purification twice, the cubosome sample exhibited a significant absorbance signal not attributable to HRP, suggesting that TA residues remaining in the supernatant caused strong false-positive readings, rendering the microBCA assay incompatible with our system.

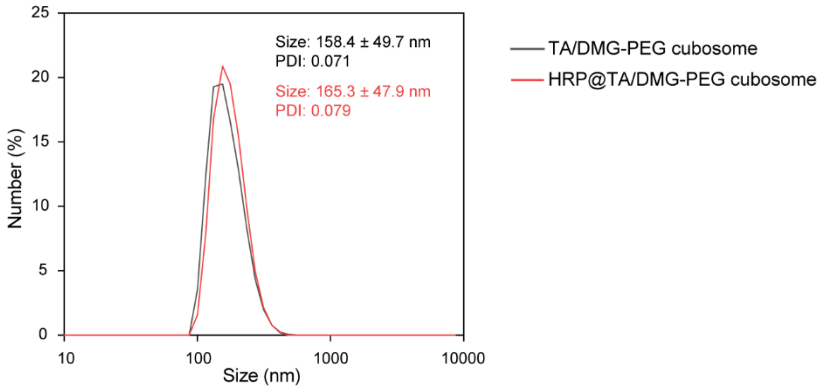

Figure S36. Size distributions of TA/DMG-PEG cubosomes before (TA/DMG-PEG cubosome) and after (HRP@TA/DMG-PEG cubosome) loading HRP.

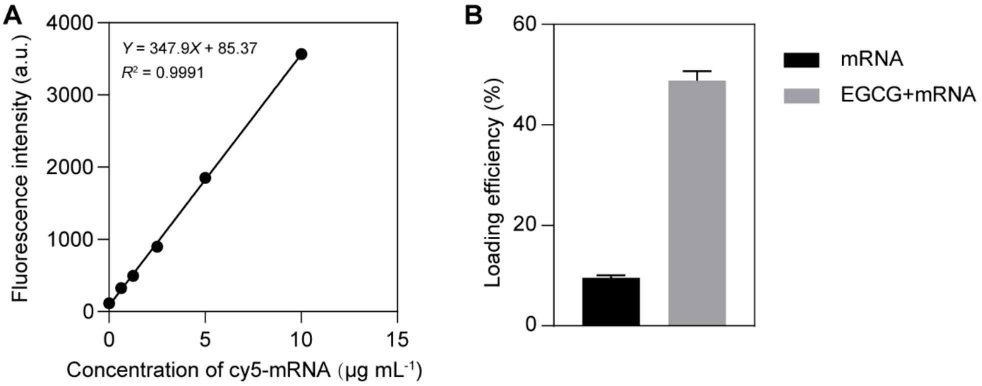

Figure S37. (**A**) Standard curve of the fluorescence intensity of cy5-mRNA of different concentrations. (**B**) mRNA loading efficiency of EGCG-Zn^{2+}/DMG-PEG cubosomes using different strategies, including incubating the cubosomes with mRNA (mRNA) and incubating the cubosomes with EGCG+mRNA (EGCG+mRNA). Data are shown as mean ± SD (*n* = 3 independent replicates).

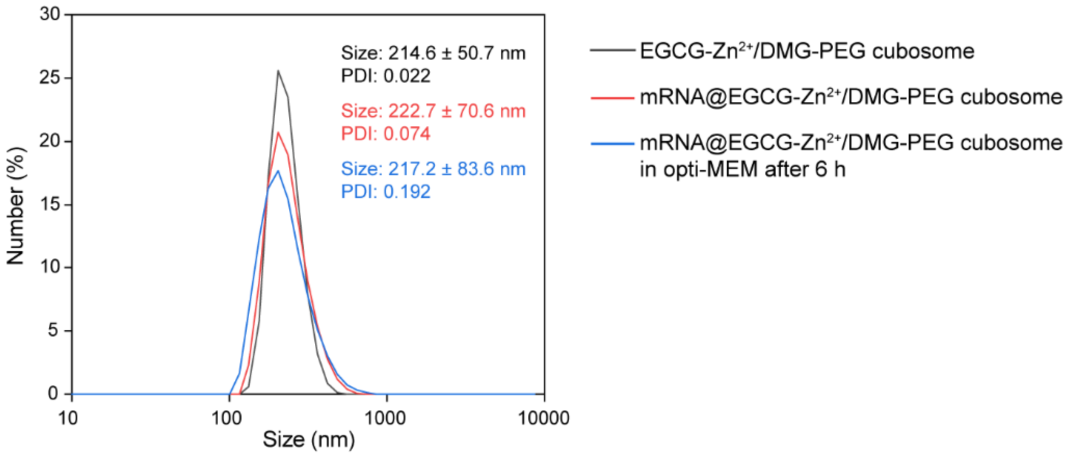

Figure S38. Size distributions of EGCG-Zn^{2+}/DMG-PEG cubosomes before and after loading with mCherry mRNA, and mRNA-loaded cubosomes after incubation in Opti-MEM for 6 h.

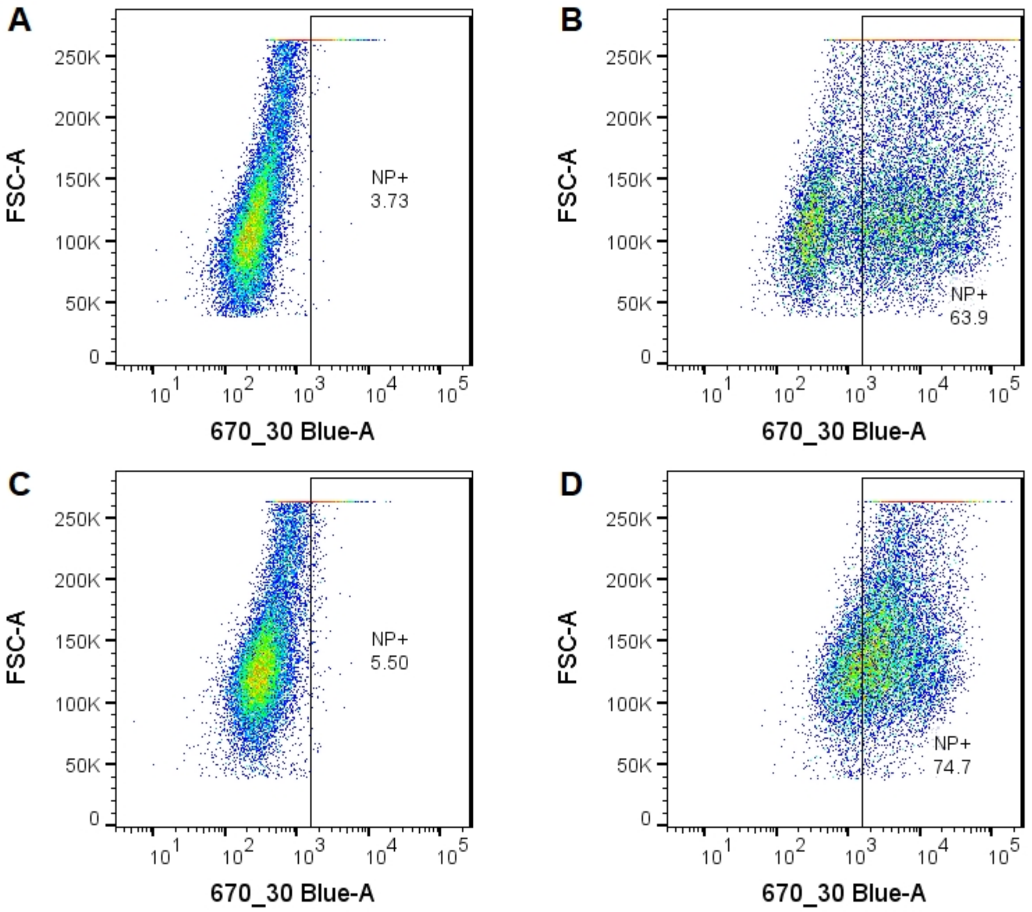

Figure S39. (**A–D**) Flow cytometry profiles of mCherry expression in HEK 293T cells treated with mCherry mRNA (**A**), mCherry mRNA-lipofectamine MessengerMAX (**B**), mCherry mRNA-EGCG (**C**), or mRNA@EGCG-Zn^{2+}/DMG-PEG cubosomes (**D**).

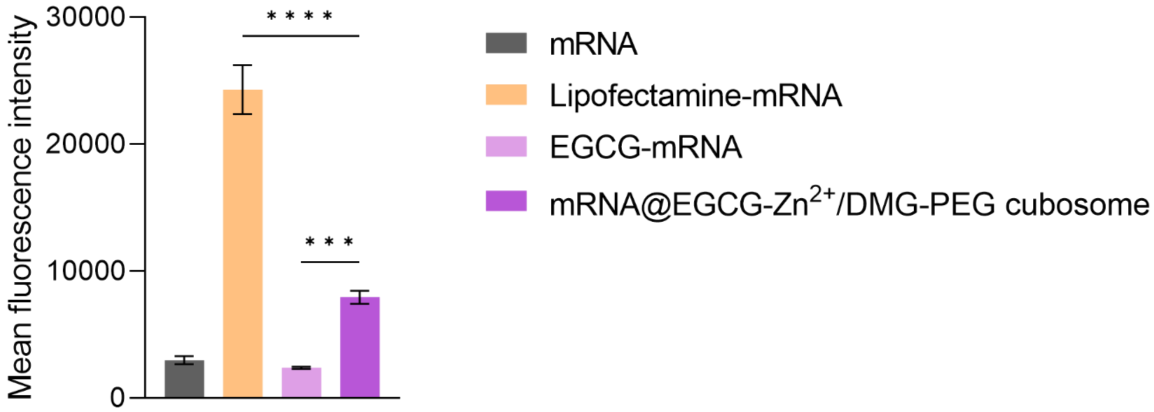

Figure S40. mCherry mRNA transfection efficiency, in terms of mean fluorescence intensity of transfected cells, in HEK 293T cells treated with mCherry mRNA (mRNA), mCherry mRNA-lipofectamine MessengerMAX (Lipofectamine-mRNA), mCherry mRNA-EGCG mixture (EGCG-mRNA), or EGCG-$Zn^{2+/}$DMG-PEG cubosomes loaded with mRNA (mRNA@EGCG-Zn^{2+}/DMG-PEG cubosome). Data are shown as mean ± SD (*n* = 3 independent replicates) and analyzed by one-way analysis of variance, ***$p < 0.001$ and ****$p < 0.0001$.

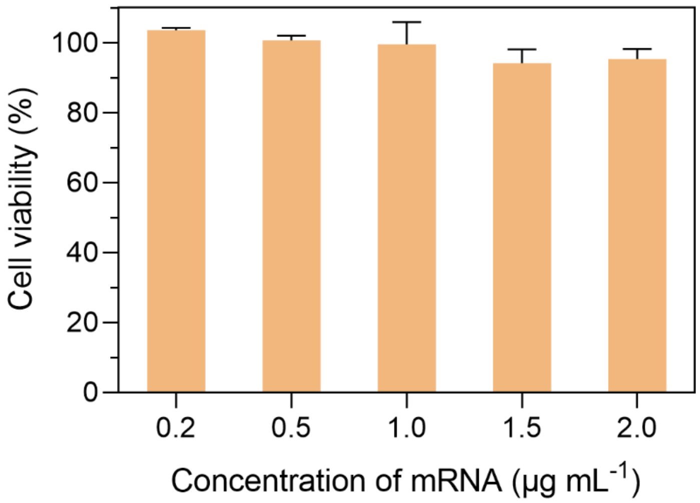

Figure S41. Viability of HEK 293T cells after treatment with mRNA@EGCG-Zn^{2+}/DMG-PEG cubosomes at different mRNA concentrations. Data are shown as mean ± SD (*n* = 3 independent replicates).

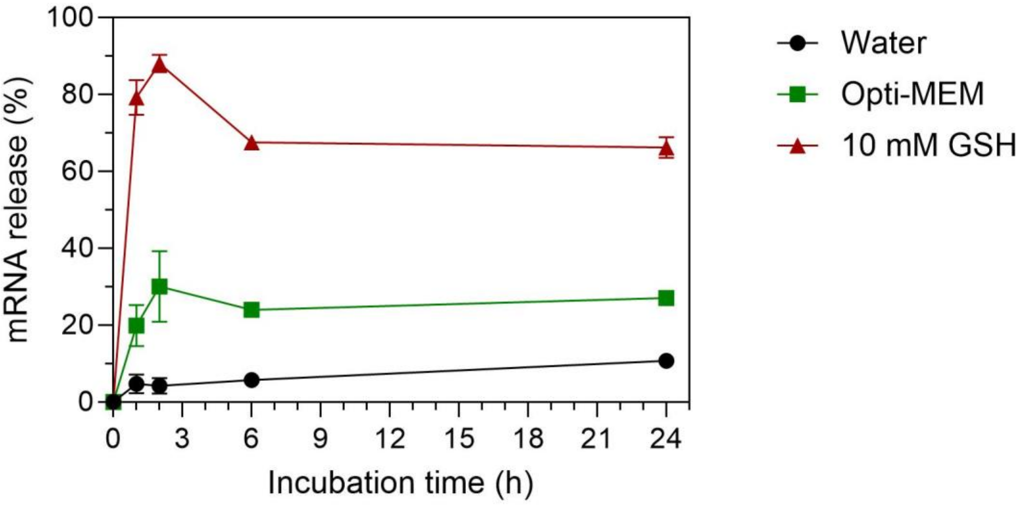

Figure S42. Time-dependent release of mRNA from EGCG-Zn^{2+}/DMG-PEG cubosomes in different media, including ultrapure water, Opti-MEM, and 10 mM GSH at 37 °C over 24 h. Data are shown as mean ± SD (n = 3 independent replicates).

Table S1. Size, polydispersity index, and ζ-potential of TA/DMG-PEG cubosomes prepared using different TA-to-DMG-PEG molar ratios at different DMG-PEG concentrations[a]

Molar ratio of TA to DMG-PEG	Size (nm)[b]	Polydispersity index[b]	ζ-Potential (mV)[c]
**2 mM DMG-PEG**			
0:1	13 ± 3	0.662	–4 ± 2
1:1 (Fig. 1C)	94 ± 28	0.072	–18 ± 1
3:1	130 ± 36	0.046	–25 ± 3
5:1 (Fig. 1D)	168 ± 40	0.016	–27 ± 1
10:1	195 ± 58	0.088	–30 ± 1
**10 mM DMG-PEG**			
1:1 (Fig. 1E)	262 ± 47	0.088	–21 ± 1

[a]The flow rate ratio of the aqueous to ethanol phase was 3:1. The total flow rate was 12 mL min^{-1}.

[b]Determined by dynamic light scattering using a Zetasizer Nano-ZS instrument.

[c]ζ-Potential measurements were performed at pH 7.4 in phosphate buffer (10 mM) using a Zetasizer Nano-ZS instrument.

Table S2. Phase information of PLC-LNPs

Sample	Phase	Lattice parameter (Å)
**TA/DMG-PEG cubosomes**		
1:1 TA:DMG-PEG (2 mM)	cubic	117.3
0.33:1 TA:DMG-PEG (2 mM)	intermediate state[a]	53.2
3:1 TA:DMG-PEG (2 mM)	cubic	117.3
5:1 TA:DMG-PEG (2 mM)	cubic	119.2
10:1 TA:DMG-PEG (2 mM)	cubic	121.2
10:1 TA:DMG-PEG (2 mM) at 4 °C for 1 year	cubic	119.6
10:1 TA:DMG-PEG (2 mM) at 22 °C for 1 year	cubic	117.5
0.33:1 TA:DMG-PEG (10 mM)	intermediate state[a]	51.2
0.5:1 TA:DMG-PEG (10 mM)	intermediate state[a]	59.9
0.6:1 TA:DMG-PEG (10 mM)	intermediate state[a] + cubic	115.5[b]
1:1 TA:DMG-PEG (10 mM)	cubic	115.5
1:1 TA:DMG-PEG (10 mM) in urea	cubic	117.4
1:1 TA:DMG-PEG (10 mM) in NaCl	cubic	115.1
2:1 TA:DMG-PEG (10 mM)	cubic	115.5
3:1 TA:DMG-PEG (10 mM)	cubic	117.3
**TA-metal ion/DMG-PEG cubosomes (different molar ratios)**		
200:1 TA:Zn^{2+}	cubic	113.5
50:1 TA:Zn^{2+}	cubic	111.6
10:1 TA:Zn^{2+}	cubic	119.6
2:1 TA:Zn^{2+}	cubic	113.5
1:1 TA: Zn^{2+}	cubic	111.6
200:1 TA:Fe^{3+}	cubic	113.5
50:1 TA:Fe^{3+}	cubic	113.5
10:1 TA:Fe^{3+}	cubic	113.5
2:1 TA:Fe^{3+}	cubic	113.5
1:1 TA:Fe^{3+}	cubic	117.5
200:1 TA:Zr^{4+}	cubic	111.6
50:1 TA:Zr^{4+}	cubic	113.5
10:1 TA:Zr^{4+}	cubic	113.5
2:1 TA:Zr^{4+}	cubic	113.5
**EGCG-metal ion/DMG-PEG cubosomes (different molar ratios)**		
200:1 EGCG:Zn^{2+}	cubic	119.6
50:1 EGCG:Zn^{2+}	cubic	119.6
10:1 EGCG:Zn^{2+}	cubic	119.6
200:1 EGCG:Fe^{3+}	cubic	119.6
50:1 EGCG:Fe^{3+}	cubic	119.6
10:1 EGCG:Fe^{3+}	cubic	121.8
200:1 EGCG:Zr^{4+}	cubic	119.6

50:1 EGCG:Zr^{4+}	cubic	119.6
10:1 EGCG:Zr^{4+}	cubic	117.5
**ALC-0159-based cubosomes**		
TA/ALC-0159	cubic	108.4
TA-Fe^{3+}/ALC-0159	cubic	106.7
**PEG-c-DMG-based cubosomes**		
TA/PEG-c-DMG	cubic	122.3
TA-Fe^{3+}/PEG-c-DMG	cubic	124.6
**DOPE-PEG-based particles**		
TA/DOPE-PEG	intermediate state[a]	N/A
TA-Fe^{3+}/DOPE-PEG	cubic	151.4
**DOPC-based particles**		
TA/DOPC	multilamellar	50.5
TA-Fe^{3+}/DOPC	hexagonal	63.0
**Cargo-loaded cubosomes**		
DiD@TA/DMG-PEG	cubic	118.0
TA/DMG-PEG + 10% cholesterol	cubic	122.3
TA/DMG-PEG + 30% cholesterol	disordered cubic	134.7
DOX@EGCG-Fe^{3+}/DMG-PEG	cubic	127.0
HRP@TA/DMG-PEG	cubic	113.5
mRNA@EGCG-Zn^{2+}/DMG-PEG	cubic	121.8
PLA@TA/DMG-PEG cubosomes (different mass ratios)		
50:1 TA:PLA	cubic	119.6
20:1 TA:PLA	cubic	117.5
5:1 TA:PLA	cubic	117.5
chitosan@TA/DMG-PEG cubosomes (different mass ratios)		
100:1 TA:chitosan	cubic	113.5
50:1 TA:chitosan	cubic	113.5
20:1 TA:chitosan	cubic	113.5
5:1 TA:chitosan	cubic	113.5

[a]Refers to the intermediate state between Type I micellar and Type I micellar cubic phase.
[b]Lattice parameter of cubosome. The lattice parameter of the multilamellar phase cannot be determined due to peak overlay.

Table S3. Details of system compositions and box dimensions for the MD simulations representing TA/lipid structures formed at different stages in the mesoscale self-assembly.

TA:lipid ratio	Molecules per cell			Number of atoms	Cubic cell side length (nm)		
	TA	lipid	H_2O		Shrinking[a]	Cooling[b]	Ambient[c]
TA–lipid seeds in initial dilute stages (full hydration: 900 water per lipid/TA)							
**0.33:1**^{fh}	25	75	90,000	286,500	–	–	14.18
**1:1**^{fh}	50	50	90,000	286,800	–	–	14.19
**3:1**^{fh}	75	25	90,000	287,100	–	–	14.20
Transient structures in intermediary stages (moderate hydration: 150 water per lipid/TA)							
**0:1**^{mh}	0	50	7,500	30,600	14.0–7.0	7.47 (0.11)	6.71
**0.02:1**^{mh}	1	50	7,650	31,224	14.0–7.5	7.55 (0.03)	6.76
**0.33:1**^{mh}	15	50	9,750	39,960	14.0–9.0	8.48 (0.24)	7.35
**1:1**^{mh}	50	50	15,000	61,800	17.0–9.5	9.41 (0.03)	8.52
**3:1**^{mh}	150	50	30,000	124,200	20.0–11.5	11.77 (0.06)	10.77
**2.7:1**^{mh}*	135	50	27,750	127,440	25.0–12.0	11.87 (0.03)	10.84
"Dry" assembled structures in final stages (low hydration: 15 water per lipid/TA)							
**0:1**	0	50	750	10,350	14.0–5.0	5.16 (0.04)	4.65
**0.02:1**	1	50	765	10,569	14.0–5.0	5.20 (0.05)	4.69
**0.33:1**	15	50	975	13,635	14.0–5.0	5.54 (0.13)	5.13
**1:1**	50	50	1,500	21,300	17.0–6.0	6.36 (0.08)	5.99
**3:1**	150	50	3,000	43,200	20.0–9.0	8.78 (0.12)	7.64
**2.7:1***	135	50	2,775	52,515	25.0–9.0	8.77 (0.10)	8.09

[a]Initial and final cell lengths for 800K *NVT* shrinking, with sides decreasing at –0.1 nm ns^{-1}.
[b]Average cell side length (± SD) for *NPT* cooling from 500–300 K at –0.5 K ns^{-1}. Taken over the initial 200 ps at 500 K and 1 atm (20 datapoints, one every 10 ps), illustrating that cell lengths deviate less than 0.55 nm when ensembles first switching from *NVT* to *NPT*.
[c]Average cell side length for ambient *NPT* simulations (303.15 K, 1 atm). Taken over the final, equilibrated 60 ns (6,000 datapoints, one every 10 ps), with SDs below 0.01 nm.
*Systems containing DMG-PEG_{2000}. All other systems are DMG-PEG_{400}.

Movie S1. Structure and dynamics of the cubosome-like system (2.7:1). (**A**) The structure and mobility of all molecular components within the assembly are depicted. DMG-PEG$_{2000}$ lipid tails and diglyceride moieties are shown in yellow, PEG chains in pink, and TA molecules in cyan. Water molecules are colored based on their occupancy in the simulation cell over the equilibrated portion (last 60 ns) of the trajectory. This coloring is derived from an average water occupancy map created using the VolMap plugin of VMD, with a voxel resolution of 1 Å^3. Red indicates positions frequently occupied over time (persistent), while blue indicates positions with lower occupancy (dynamic channels). The trajectory was smoothed to remove high-frequency motion, enhancing the clarity of the occupancy data. Note that individual water molecules change color over time as they move between different voxel positions. Hydrogen atoms on all molecules are omitted for clarity. (**B**) Focuses exclusively on the water molecules.

Supporting References

1. J. Song, C. Cortez-Jugo, S. J. Shirbin, Z. Lin, S. Pan, G. G. Qiao, F. Caruso, *Adv. Funct. Mater.* **2021**, *32*, 2107341.
2. R. Qaqish, M. Amiji, *Carbohydr. Polym.* **1999**, *38*, 99.
3. M. Huang, Z. Ma, E. Khor, L.-Y. Lim, *Pharm. Res.* **2002**, *19*, 1488.
4. F.-L. Mi, Y.-Y. Wu, Y.-L. Chiu, M.-C. Chen, H.-W. Sung, S.-H. Yu, S.-S. Shyu, M.-F. Huang, *Biomacromolecules* **2007**, *8*, 892.
5. M. Faria, M. Björnmalm, K. J. Thurecht, S. J. Kent, R. G. Parton, M. Kavallaris, A. P. R. Johnston, J. J. Gooding, S. R. Corrie, B. J. Boyd, P. Thordarson, A. K. Whittaker, M. M. Stevens, C. A. Prestidge, C. J. H. Porter, W. J. Parak, T. P. Davis, E. J. Crampin, F. Caruso, *Nat. Nanotechnol.* **2018**, *13*, 777–785.
6. M. Sonora, L. Martinez, S. Pantano, M. R. Machado, *J. Chem. Inf. Model.* **2021**, *61*, 408–442.
7. L. Martínez, R. Andrade, E. G. Birgin, J. M. Martínez, *J. Comput. Chem.* **2009**, *30*, 2157–2164.
8. S. Park, Y. K. Choi, S. Kim, J. Lee, W. Im, *J. Chem. Inf. Model.* **2021**, *61*, 5192–5202.
9. W. Humphrey, A. Dalke, K. Schulten, *J. Mol. Graphics* **1996**, *14*, 33–38.
10. J. C. Phillips, D. J. Hardy, J. D. C. Maia, J. E. Stone, J. V. Ribeiro, R. C. Bernardi, R. Buch, G. Fiorin, J. Hénin, W. Jiang, R. McGreevy, M. C. R. Melo, B. K. Radak, R. D. Skeel, A. Singharoy, Y. Wang, B. Roux, A. Aksimentiev, Z. Luthey-Schulten, L. V. Kalé, K. Schulten, C. Chipot, E. Tajkhorshid, *J. Chem. Phys.* **2020**, *153*, 044130.
11. J. B. Klauda, R. M. Venable, J. A. Freites, J. W. O'Connor, D. J. Tobias, C. Mondragon-Ramirez, I. Vorobyov, A. D. MacKerell, Jr., R. W. Pastor, *J. Phys. Chem. B* **2010**, *114*, 7830–7843.
12. K. Vanommeslaeghe, E. Hatcher, C. Acharya, S. Kundu, S. Zhong, J. Shim, E. Darian, O. Guvench, P. Lopes, I. Vorobyov, A. D. Mackerell, Jr., *J. Comput. Chem.* **2010**, *31*, 671–690.
13. O. Guvench, S. N. Greene, G. Kamath, J. W. Brady, R. M. Venable, R. W. Pastor, A. D. Mackerell, Jr., *J. Comput. Chem.* **2008**, *29*, 2543–2564.
14. W. L. Jorgensen, J. Chandrasekhar, J. D. Madura, R. W. Impey, M. L. Klein, *J. Chem. Phys.* **1983**, *79*, 926–935.
15. A. D. MacKerell, Jr., D. Bashford, M. Bellott, R. L. Dunbrack, Jr., J. D. Evanseck, M. J. Field, S. Fischer, J. Gao, H. Guo, S. Ha, D. Joseph-McCarthy, L. Kuchnir, K. Kuczera, F. T. K. Lau, C. Mattos, S. Michnick, T. Ngo, D. T. Nguyen, B. Prodhom, W. E. Reiher, B. Roux, M. Schlenkrich, J. C. Smith, R. Stote, J. Straub, M. Watanabe, J. Wiórkiewicz-Kuczera, D. Yin, M. Karplus, *J. Phys. Chem. B* **1998**, *102*, 3586–3616.
16. J. V. Vermaas, L. Petridis, J. Ralph, M. F. Crowley, G. T. Beckham, *Green Chem.* **2019**, *21*, 109–122.
17. Y. Ishii, N. Matubayasi, G. Watanabe, T. Kato, H. Washizu, *Sci. Adv.* **2021**, *7*, eabf0669.
18. T. Darden, D. York, L. Pedersen, *J. Chem. Phys.* **1993**, *98*, 10089–10092.
19. G. J. Martyna, D. J. Tobias, M. L. Klein, *J. Chem. Phys.* **1994**, *101*, 4177–4189.
20. S. E. Feller, Y. Zhang, R. W. Pastor, B. R. Brooks, *J. Chem. Phys.* **1995**, *103*, 4613–4621.
21. Y. Wang, A. Kiziltas, P. Blanchard, T. R. Walsh, *Comput. Phys. Commun.* **2021**, *266*, 108032.
22. N. Michaud-Agrawal, E. J. Denning, T. B. Woolf, O. Beckstein, *J. Comput. Chem.* **2011**, *32*, 2319–2327.
23. B. M. H. Bruininks, T. A. Wassenaar, I. Vattulainen, *J. Chem. Inf. Model.* **2023**, *63*, 3448–3452.
24. S. Mann, *Nat. Mater.* **2009**, *8*, 781–792.

Checklist
Minimum Information Reporting in Bio–Nano Experimental Literature

The MIRIBEL guidelines were introduced here: https://doi.org/10.1038/s41565-018-0246-4
The development of these guidelines was led by the ARC Centre of Excellence in Convergent Bio-Nano Science and Technology: https://www.cbns.org.au/. Any updates or revisions to this document will be made available here: http://doi.org/10.17605/OSF.IO/SMVTF. This document is made available under a CC-BY 4.0 license: https://creativecommons.org/licenses/by/4.0/.
The MIRIBEL guidelines were developed to facilitate reporting and dissemination of research in bio–nano science. Their development was inspired by various similar efforts:

• MIAME (microarray experiments): Nat. Genet. 29 (2001), 365; http://doi.org/10.1038/ng1201365
• MIRIAM (biochemical models): Nat. Biotechnol. 23 (2005) 1509; http://doi.org/10.1038/nbt1156
• MIBBI (biology/biomedicine): Nat. Biotechnol. 26 (2008) 889; http://doi.org/10.1038/nbt.1411
• MIGS (genome sequencing): Nat. Biotechnol. 26 (2008) 541; http://doi.org/10.1038/nbt1360
• MIQE (quantitative PCR): Clin. Chem. 55 (2009) 611; http://doi.org/10.1373/clinchem.2008.112797
• ARRIVE (animal research): PLOS Biol. 8 (2010) e1000412; http://doi.org/10.1371/journal.pbio.1000412
• Nature's reporting standards:
o Life science: https://www.nature.com/authors/policies/reporting.pdf; e.g., Nat. Nanotechnol. 9 (2014) 949; http://doi.org/10.1038/nnano.2014.287
o Solar cells: https://www.nature.com/authors/policies/solarchecklist.pdf; e.g., Nat. Photonics 9 (2015) 703; http://doi.org/10.1038/nphoton.2015.233
o Lasers: https://www.nature.com/authors/policies/laserchecklist.pdf; e.g., Nat. Photonics 11 (2017) 139; http://doi.org/10.1038/nphoton.2017.28
• The "TOP guidelines": e.g., Science 352 (2016) 1147; http://doi.org/10.1126/science.aag2359

Similar to many of the efforts listed above, the parameters included in this checklist are **not** intended to be definitive requirements; instead they are intended as 'points to be considered', with authors themselves deciding which parameters are—and which are not—appropriate for their specific study.
This document is intended to be a living document, which we propose is revisited and amended annually by interested members of the community, who are encouraged to contact the authors of this document.
Parts of this document were developed at the annual International Nanomedicine Conference in Sydney, Australia: http://www.oznanomed.org/, which will continue to act as a venue for their review and development, and interested members of the community are encouraged to attend.
After filling out the following pages, this checklist document can be attached as a "Supporting Information" document during submission of a manuscript to inform Editors and Reviewers (and eventually readers) that all points of MIRIBEL have been considered.

Table 1. Material characterization*

**Question**	**Yes**	**No**
1.1 Are "**best reporting practices**" **available** for the nanomaterial used? For examples, see *Chem. Mater.* **28** (2016) 3535; http://doi.org/10.1021/acs.chemmater.6b01854 and *Chem. Mater.* **29** (2017) 1; http://doi.org/10.1021/acs.chemmater.6b05235	Not applicable	
1.2 If they are available, **are they used**? If not available, ignore this question and proceed to the next one.		
1.3 Are extensive and clear instructions reported detailing all steps of **synthesis** and the resulting **composition** of the nanomaterial? For examples, see *Chem. Mater.* **26** (2014) 1765; http://doi.org/10.1021/cm500632c, and *Chem. Mater.* **26** (2014) 2211; http://doi.org/10.1021/cm5010449. Extensive use of photos, images, and videos are strongly encouraged. For example, see *Chem. Mater.* **28** (2016) 8441; http://doi.org/10.1021/acs.chemmater.6b04639	✓	
1.4 Is the **size** (or **dimensions**, if non-spherical) and **shape of** the nanomaterial reported?	✓	
1.5 Is the **size dispersity** or **aggregation** of the nanomaterial reported?	✓	
1.6 Is the **zeta potential** of the nanomaterial reported?	✓	
1.7 Is the **density (mass/volume)** of the nanomaterial reported?	✓	
1.8 Is the amount of any **drug loaded** reported? 'Drug' here broadly refers to functional cargos (e.g., proteins, small molecules, nucleic acids).	✓	
1.9 Is the **targeting performance** of the nanomaterial reported, including **amount** of ligand bound to the nanomaterial if the material has been functionalised through addition of targeting ligands?	Not applicable	
1.10 Is the **label signal** per nanomaterial/particle reported? For example, fluorescence signal per particle for fluorescently labelled nanomaterials.	Not applicable	
1.11 If a material property not listed here is varied, has it been **quantified**?	Not applicable	
1.12 Were characterizations performed in a **fluid mimicking biological conditions**?	✓	
1.13 Are details of how these parameters were **measured/estimated** provided?	✓	
Explanation for **No** (if needed): No targeting experiments were carried out.		

Table 2. Biological characterization*

Question	Yes	No
2.1 Are **cell seeding details**, including **number of cells plated, confluency at start of experiment,** and **time between seeding and experiment** reported?	✓	
2.2 If a standardised cell line is used, are the **designation and source** provided?	✓	
2.3 Is the **passage number** (total number of times a cell culture has been subcultured) known and reported?	✓	
2.4 Is the last instance of **verification of cell line** reported? If no verification has been performed, is the time passed and passage number since acquisition from trusted source (e.g., ATCC or ECACC) reported? For information, see *Science* **347** (2015) 938; http://doi.org/10.1126/science.347.6225.938	✓	
2.5 Are the results from **mycoplasma testing** of cell cultures reported?	✓	
2.6 Is the **background signal of cells**/**tissue** reported? (E.g., the fluorescence signal of cells without particles in the case of a flow cytometry experiment.)	✓	
2.7 Are **toxicity studies** provided to demonstrate that the material has the expected toxicity, and that the experimental protocol followed does not?	✓	
2.8 Are details of media preparation (**type of media, serum,** any **added antibiotics**) provided?	✓	
2.9 Is a **justification of the biological model** used provided? For examples for cancer models, see *Cancer Res.* **75** (2015) 4016; http://doi.org/10.1158/0008-5472.CAN-15-1558, and *Mol. Ther.* **20** (2012) 882; http://doi.org/10.1038/mt.2012.73, and *ACS Nano* **11** (2017) 9594; http://doi.org/10.1021/acsnano.7b04855	✓	
2.10 Is characterization of the **biological fluid** (*ex vivo*/*in vitro*) reported? For example, when investigating protein adsorption onto nanoparticles dispersed in blood serum, pertinent aspects of the blood serum should be characterised (e.g., protein concentrations and differences between donors used in study).	Not applicable	
2.11 For **animal experiments**, are the ARRIVE guidelines followed? For details, see *PLOS Biol.* **8** (2010) e1000412; http://doi.org/10.1371/journal.pbio.1000412	Not applicable	
Explanation for **No** (if needed): No protein corona or animal experiments were included in this study.		

Table 3. Experimental details*

Question	Yes	No
3.1 For cell culture experiments: are **cell culture dimensions** including **type of well**, **volume of added media**, reported? Are cell types (i.e.; adherent vs suspension) and **orientation** (if non-standard) reported?	✓	
3.2 Is the **dose of material administered** reported? This is typically provided in nanomaterial mass, volume, number, or surface area added. Is sufficient information reported so that regardless of which one is provided, the other dosage metrics can be calculated (i.e. using the dimensions and density of the nanomaterial)?	✓	
3.3 For each type of imaging performed, are details of how **imaging** was performed provided, including details of **shielding, non-uniform image processing**, and any **contrast agents** added?	✓	
3.4 Are details of how the dose was administered provided, including **method of administration, injection location, rate of administration,** and details of **multiple injections**?	Not applicable	
3.5 Is the methodology used to **equalise dosage** provided?	✓	
3.6 Is the **delivered dose** to tissues and/or organs (in vivo) reported, as % injected dose per gram of tissue (%ID g^{-1})?	Not applicable	
3.7 Is **mass of each organ/tissue measured** and **mass of material** reported?	Not applicable	
3.8 Are the **signals of cells/tissues with nanomaterials** reported? For instance, for fluorescently labelled nanoparticles, the total number of particles per cell or the fluorescence intensity of particles + cells, at each assessed timepoint.	✓	
3.9 Are **data analysis details**, including **code used** for analysis provided?	✓	
3.10 Is the **raw data** or **distribution of values** underlying the reported results provided? For examples, see *R. Soc. Open Sci.* **3** (2016) 150547; http://doi.org/10.1098/rsos.150547, https://opennessinitiative.org/making-your-data-public/, http://journals.plos.org/plosone/s/data-availability, and https://www.nature.com/sdata/policies/repositories	Not applicable	
Explanation for **No** (if needed): No animal experiments were included in this study.		
